# *Legionella pneumophila* usurps host cell lipids for vacuole expansion and bacterial growth

**DOI:** 10.1371/journal.ppat.1011996

**Published:** 2024-02-22

**Authors:** Soma Ghosh, Saumya Bandyopadhyay, Danielle M. Smith, Sangeeta Adak, Clay F. Semenkovich, Laszlo Nagy, Michael J. Wolfgang, Tamara J. O’Connor

**Affiliations:** 1 Department of Biological Chemistry, The Johns Hopkins University School of Medicine, Baltimore, Maryland, United States of America; 2 Division of Endocrinology, Metabolism and Lipid Research, Washington University School of Medicine, St. Louis, Missouri, United States of America; 3 Department of Medicine, Institute for Fundamental Biomedical Research, Johns Hopkins All Children’s Hospital, St. Petersburg, Florida, United States of America; Purdue University, UNITED STATES

## Abstract

Vacuolar pathogens reside in membrane-bound compartments within host cells. Maintaining the integrity of this compartment is paramount to bacterial survival and replication as it protects against certain host surveillance mechanisms that function to eradicate invading pathogens. Preserving this compartment during bacterial replication requires expansion of the vacuole membrane to accommodate the increasing number of bacteria, and yet, how this is accomplished remains largely unknown. Here, we show that the vacuolar pathogen *Legionella pneumophila* exploits multiple sources of host cell fatty acids, including inducing host cell fatty acid scavenging pathways, in order to promote expansion of the replication vacuole and bacteria growth. Conversely, when exogenous lipids are limited, the decrease in host lipid availability restricts expansion of the replication vacuole membrane, resulting in a higher density of bacteria within the vacuole. Modifying the architecture of the vacuole prioritizes bacterial growth by allowing the greatest number of bacteria to remain protected by the vacuole membrane despite limited resources for its expansion. However, this trade-off is not without risk, as it can lead to vacuole destabilization, which is detrimental to the pathogen. However, when host lipid resources become extremely scarce, for example by inhibiting host lipid scavenging, *de novo* biosynthetic pathways, and/or diverting host fatty acids to storage compartments, bacterial replication becomes severely impaired, indicating that host cell fatty acid availability also directly regulates *L*. *pneumophila* growth. Collectively, these data demonstrate dual roles for host cell fatty acids in replication vacuole expansion and bacterial proliferation, revealing the central functions for these molecules and their metabolic pathways in *L*. *pneumophila* pathogenesis.

## Author summary

The pathogenesis of bacteria depends on their ability to avoid detection and killing by the host immune system while simultaneously competing with the host for nutrients. Here, we demonstrate that host cell fatty acids are paramount to the intracellular survival and replication of the bacterial pathogen *Legionella*. *Legionella* infection induces host cell fatty acid scavenging allowing for critical expansion of its replication vacuole, which protects against recognition by host surveillance pathways, and mediates robust bacterial proliferation. However, if host cell fatty acid availability becomes limited, vacuole membrane expansion is constrained, often leading to vacuole destabilization, and bacterial growth is restricted. Our findings reveal a central role for host cell fatty acids and their metabolic pathways in bacterial pathogenesis, whereby both the host and the pathogen define a gradient of available host fatty acids that can be manipulated by either organism to their benefit.

## Introduction

Lipids perform fundamental roles in a variety of cellular processes, including energy production and storage, immune signaling and as structural components of cellular membranes. In mammalian cells, membranes are composed of a combination of sterols, predominantly cholesterol, and phospholipids and sphingolipids, comprised of fatty acids covalently linked to a polar head group [1]. The availability, chemical composition and relative abundance of these molecules have profound effects on membrane production and its biophysical properties such as membrane tension, fluidity and shape, which can greatly impact function [2]. Thus, the ability of intracellular pathogens to modulate lipid metabolism could have profound effects on the outcome of infection. While the link between host cell cholesterol and the remodeling and trafficking of pathogen phagosomes is well-documented [3–6], much less is known about the direct roles of host cell fatty acids in the generation and/or maintenance of pathogen replication compartments.

Many bacterial pathogens that grow within host cells do so in specialized, membrane-bound compartments [7]. While the nature of the replication vacuole varies between pathogens, maintaining its integrity throughout the infection cycle is critical for pathogenesis, as it provides protection against host surveillance systems that detect and eliminate pathogens [8–10]. Despite the crucial role of vacuole stability, the mechanisms responsible for its preservation are poorly understood.

*Legionella pneumophila* are intracellular bacterial pathogens that replicate within alveolar macrophages [11] causing pneumonia [12,13]. Upon entering the host cell, *L*. *pneumophila* remodel their phagosomes into replication-permissive compartments called the *Legionella*-containing vacuole (LCV) [14,15]. The generation of the LCV through the hijacking of host ER-derived vesicles and interacting with ER tubules [14–21] takes 4–6 hours to complete. This process is mediated by a Type IVb secretion system called Dot/Icm that translocates bacterial proteins, termed effectors, into the host cell [22]. Once the LCV is established, the bacteria begin to replicate, resulting in 50–100 bacteria per host cell. Since proliferation occurs within host cells, bacterial growth depends on the host cell for nutrients [23–25]. Roughly 16–18 hours after infecting the host cell, the bacteria exit, killing the host cell in the process.

Electron microscopy of *L*. *pneumophila*-infected cells show that the LCV membrane is tightly juxtaposed to the bacteria, outlining the contour of the bacteria in three-dimensional space [15,16,26,27]. As a result, bacterial replication requires expansion of the LCV membrane while maintaining LCV integrity [28], suggesting a critical requirement for host cell lipids in this process. In support of this, *L*. *pneumophila* infection activates mTORC1 and SREBP [28], pivotal regulators of host lipid synthesis, and correspondingly, inhibition of mTORC1 or SREBP destabilizes the LCV, restricting bacterial growth [28]. More specifically, the cholesterol biosynthesis branch of SREBP-controlled lipid production has been shown to be important for *L*. *pneumophila* intracellular replication, linking this membrane constituent to the abundance of *L*. *pneumophila* within host cells [29]. Here, we show that in addition to cholesterol, *L*. *pneumophila* relies on host cell fatty acids for its intracellular growth, hijacking host cellular resources to fuel LCV membrane expansion and bacterial growth.

## Results

### *L*. *pneumophila* exploit multiple host lipid resources for their intracellular replication

**Fig 1:**
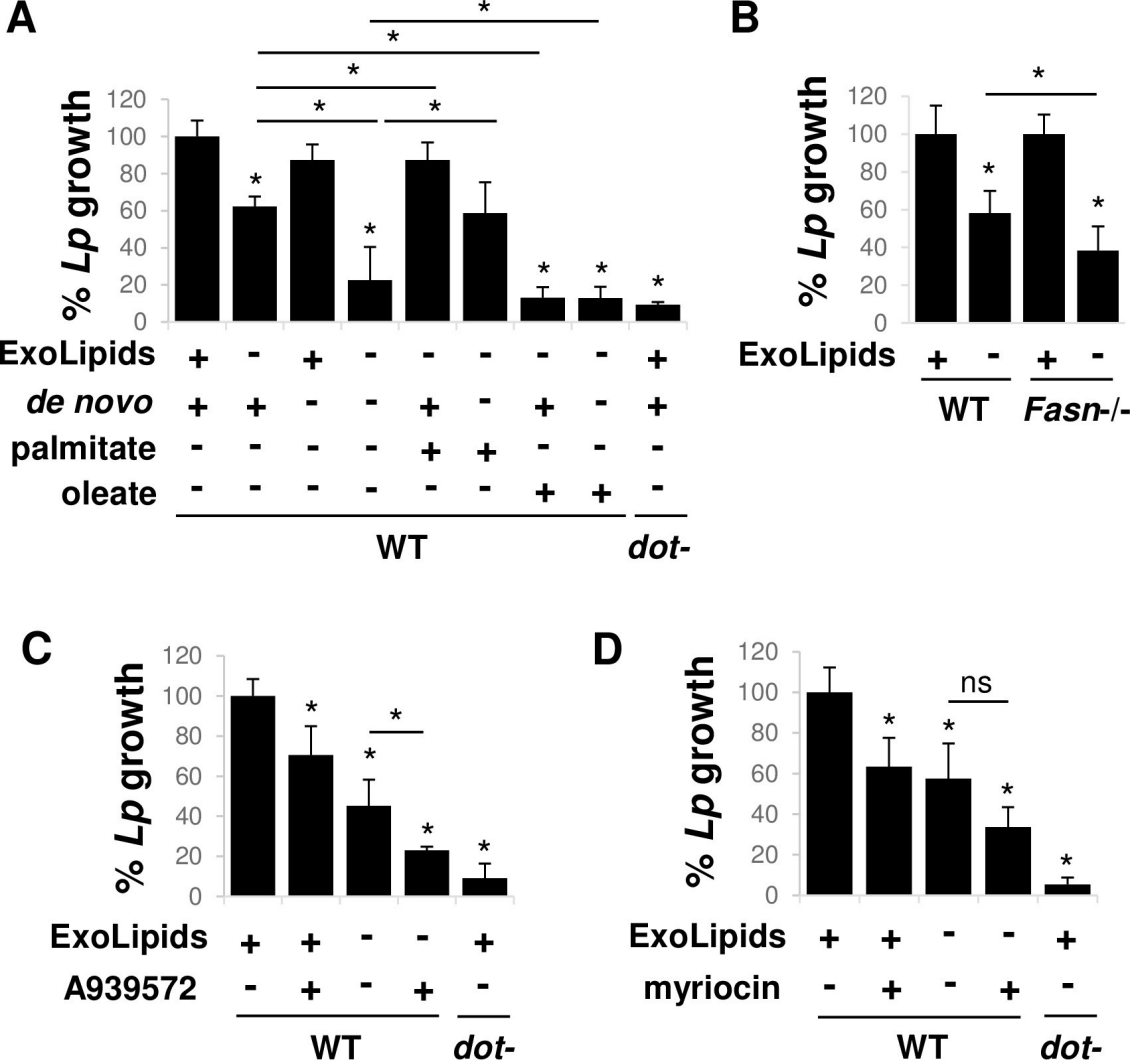
Host cell fatty acids are required for *L*. *pneumophila* intracellular replication. **A)** Limiting exogenous lipids and *de novo* lipid biosynthesis reduces *L*. *pneumophila* replication within host cells. Primary bone marrow-derived A/J mouse macrophages were infected with *L*. *pneumophila* (*Lp*) in the presence (+) or absence (-) of serum (exogenous lipids (ExoLipids)) and/or in the absence of *de novo* lipid synthesis (*de novo*) by treating cells with the SREBP1 inhibitor fatostatin, and/or the presence or absence of the fatty acid palmitate or oleate. Bacterial replication was quantified based on recovered colony forming units (cfus) from host cell lysates at 24 hrs, equivalent to a single round of infection, and normalized to wild type (WT) bacteria in untreated host cells by the number of bacteria at 1 hpi. *dot*-, replication deficient, avirulent strain. **B)** Loss of fatty acid synthase restricts *L*. *pneumophila* intracellular growth. Primary bone marrow-derived macrophages from WT and *Fasn*-/- mice were infected with *L*. *pneumophila* in the presence and absence of exogenous lipids and bacterial replication was quantified as in A). **C)** Impaired production of unsaturated fatty acids within host cells limits *L*. *pneumophila* intracellular replication. Primary A/J mouse macrophages were infected with *L*. *pneumophila* in the presence or absence of exogenous lipids and the stearoyl-CoA desaturase-1 inhibitor A395372 and bacterial replication was quantified as in A). **D)** *L*. *pneumophila* intracellular growth depends on host cell sphingolipids precursors. Primary A/J mouse macrophages were infected with *L*. *pneumophila* in the presence or absence of exogenous lipids and the serine palmitoyltransferase inhibitor myriocin and bacterial replication was quantified as in A). **A-D)** Data are the mean ± standard deviation of 3 biological replicates consisting of 3 technical replications each. *, Student’s t-test p value <0.05 compared to WT bacteria in untreated host cells, unless otherwise indicated. ns, not significant.

There are three main sources of lipids within mammalian host cells; exogenous lipids derived from serum, *de novo* lipid biosynthesis, and stored lipids in the form of lipid droplets. Given the link between regulators of host cell lipid metabolism and *L*. *pneumophila* pathogenesis [28], we examined the importance of each of these three sources for *L*. *pneumophila* replication. Consistent with data from Ivanov and colleagues [28], we observed reduced *L*. *pneumophila* replication in macrophages cultured in the absence of exogenous lipids (Fig 1A). Furthermore, we found that while inhibiting *de novo* lipid biosynthesis (sterols and fatty acids) using the SREBP inhibitor fatostatin did not impair *L*. *pneumophila* growth on its own (Fig 1A), inhibiting *de novo* lipid biosynthesis in the absence of exogeneous lipids further reduced the number of wild type (WT) bacteria to near that of a non-replicating, *dot*- avirulent strain (Fig 1A). These intracellular growth defects could be rescued by supplementing the host cell culture medium at 1 hour post infection (hpi) with palmitate, a 16-carbon saturated fatty acid (Fig 1A). Collectively, these results indicate that the fatty acid component of serum lipids plays a central role in supporting *L*. *pneumophila* replication. In contrast, supplementing host cells with oleate, an 18-carbon mono-unsaturated fatty acid was unable to rescue the *L*. *pneumophila* growth defect observed in the absence of exogenous lipids, and instead reduced bacterial numbers to that of a *dot*- strain (Fig 1A). These results indicated that oleate aggravates the *L*. *pneumophila* growth defect observed in the absence of exogenous lipids, and distinct differences between the effects of palmitate and oleate on *L*. *pneumophila* intracellular replication. However, this was not due to their differential effects on host *de novo* lipogenesis (S1 Fig) and is examined further below.

Confirming a role for *de novo* fatty acid biosynthesis, we observed a reduction in *L*. *pneumophila* growth in primary macrophages isolated from mice with a myeloid-specific deletion of fatty acid synthase (FASN) (*Fasn*-/-) [30] when cultured in the absence of exogenous lipids (Fig 1B). FASN produces long chain saturated fatty acids, that can then be elongated and desaturated, all of which can then be incorporated into complex lipids such as phospholipids and sphingolipids. Notably, chemical inhibition of host cell palmitate utilization pathways using A939572, which inhibits fatty acid delta-9 desaturation by stearoyl-CoA desaturase-1 (SCD1) or using myriocin, which inhibits sphingolipid production by serine palmitoyltransferase (SPT), similarly restricted *L*. *pneumophila* replication (Fig 1C and 1D). The inhibition of *L*. *pneumophila* replication under each of the host cell conditions tested was not due to an effect on host cell viability, as no difference in host cell survival (S2A Fig) or cellular respiration (S2B Fig) was observed. Furthermore, our results are supported by the identification of several genes involved in lipid synthesis and/or metabolism in a genetic screen for host genes that support *L*. *pneumophila* growth, including FASN, several enzymes involved in fatty acid elongation, SPT and ceramide synthase that functions downstream of SPT [31]. Collectively, our data indicate that the fatty acid component of serum lipids and the fatty acid synthesis branch of SREBP-dependent lipid biosynthesis play a central role in *L*. *pneumophila* pathogenesis. More specifically, these results demonstrate that *L*. *pneumophila* replication depends on host cell fatty acids, for which the bacteria exploit at least two host cellular sources of lipids, exogenous lipids and *de novo* lipid biosynthesis.

**Fig 2:**
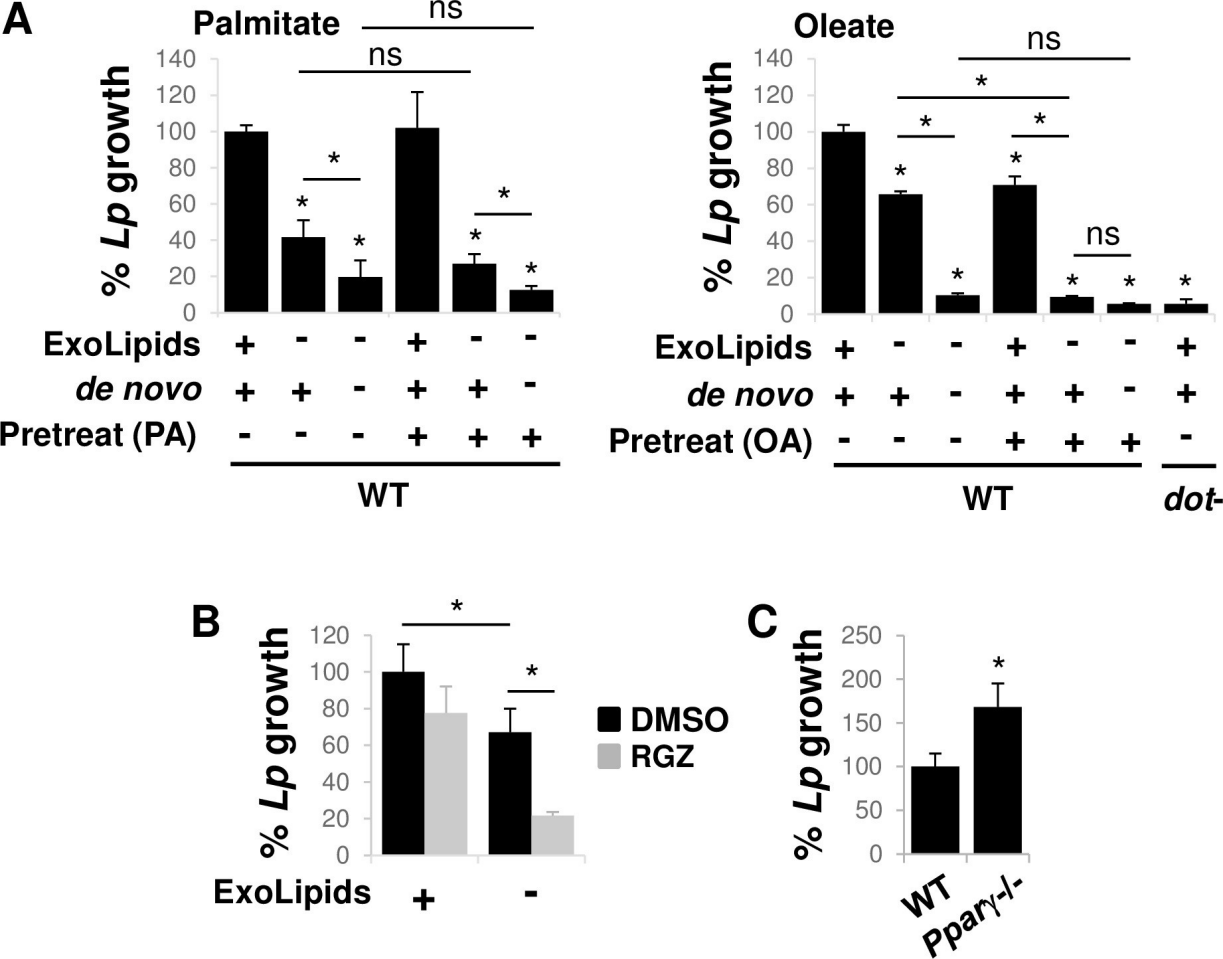
Inducing host cell lipid storage restricts *L*. *pneumophila* replication. **A)** Lipid droplets are not sufficient to rescue *L*. *pneumophila* replication when host lipid resources are limited. Primary bone marrow-derived A/J murine macrophages were pre-treated with palmitate (left panel) or oleate (right panel) for 18 hrs to induce lipid droplet formation (S3 Fig). Cells were then rinsed and infected with *L*. *pneumophila* (*Lp*) in the presence (+) or absence (-) of exogenous lipids (ExoLipids) by culturing cells in the absence of serum, or the absence of *de novo* lipid synthesis (*de novo*) by treating cells with fatostatin. The number of bacteria at 24 hpi was enumerated based on recovered cfus, normalizing to wild type (WT) bacteria in untreated host cells by bacterial cfus at 1 hpi. *dot*-, replication deficient strain. **B)** Activation of host cell PPARγ impairs *L*. *pneumophila* replication. Primary A/J murine macrophages were infected with *L*. *pneumophila* in the presence of absence of rosiglitazone, an activator of PPARγ and bacterial numbers at 24 hpi was determined as in A). DMSO (dimethyl sulfoxide), vehicle control. **C)** Loss of PPARγ enhances *L*. *pneumophila* intracellular replication. Immortalized primary macrophages isolated from WT or *Ppar*γ-/- C57BL/6 mice were infected with *L*. *pneumophila* and bacterial replication was measured as in A). A-D) Data are the mean of 3–4 biological replicates, consisting of 3 technical replicates each. *, Student’s t-test p < 0.05 relative to untreated condition (A,C) or WT cells (B) unless otherwise indicated. ns, not significant.

In addition to exogenous and *de novo* synthesized lipids, lipids stored in lipid droplets could potentially serve as an additional fatty acid resource for *L*. *pneumophila*. To test the ability of lipid droplets to function in this capacity, we first induced the production of lipid droplets by pre-treating macrophages with palmitate or oleate, which led to an increase in the size, and for oleate, the abundance of lipid droplets within the cells (S3 Fig). Next, macrophages were rinsed to remove excess fatty acids and challenged with *L*. *pneumophila* in the absence of exogenous lipids or in the absence of both exogenous lipids and *de novo* lipid synthesis. Despite palmitate supplementation rescuing *L*. *pneumophila* growth when added at the beginning of the infection (Fig 1A), inducing lipid droplets with palmitate prior to infection was unable to restore *L*. *pneumophila* replication (Fig 2A, left panel). Similarly, oleate-stimulated lipid droplets were also unable to restore *L*. *pneumophila* replication (Fig 2A, right panel). In contrast, pre-treating macrophages with oleate impaired *L*. *pneumophila* replication even in the presence of other fatty acid resources, and in the absence of exogenous lipids, reduced the number of bacteria to that observed when cells were infected with an avirulent *dot*- strain (Fig 2A, right panel). These results suggested that stored lipids are unable to serve as an alternate lipid resource for *L*. *pneumophila* replication.

To confirm the idea that stored lipids are not readily accessible to *L*. *pneumophila*, we assayed *L*. *pneumophila* growth under conditions with increased or decreased host cell lipid storage. Increased lipid storage was induced by chemically activating the transcription factor PPARγ [32], a central regulator of lipid droplet formation in macrophages [33], using rosiglitazone [34]. Decreased host cell lipid storage was achieved using immortalized primary murine *Pparγ*-/- macrophages [35]. Consistent with the data on lipid droplets, inducing host cell fatty acid storage exacerbated the *L*. *pneumophila* intracellular growth defect observed in the absence of exogenous lipids (Fig 2B) and repressing host lipid storage increased *L*. *pneumophila* replication (Fig 2C). Collectively these results suggested that diverting host cell lipids to storage compartments is detrimental to *L*. *pneumophila*, most likely by limiting access to host lipid resources.

### *L*. *pneumophila* infection drives host cell lipid scavenging

**Fig 3:**
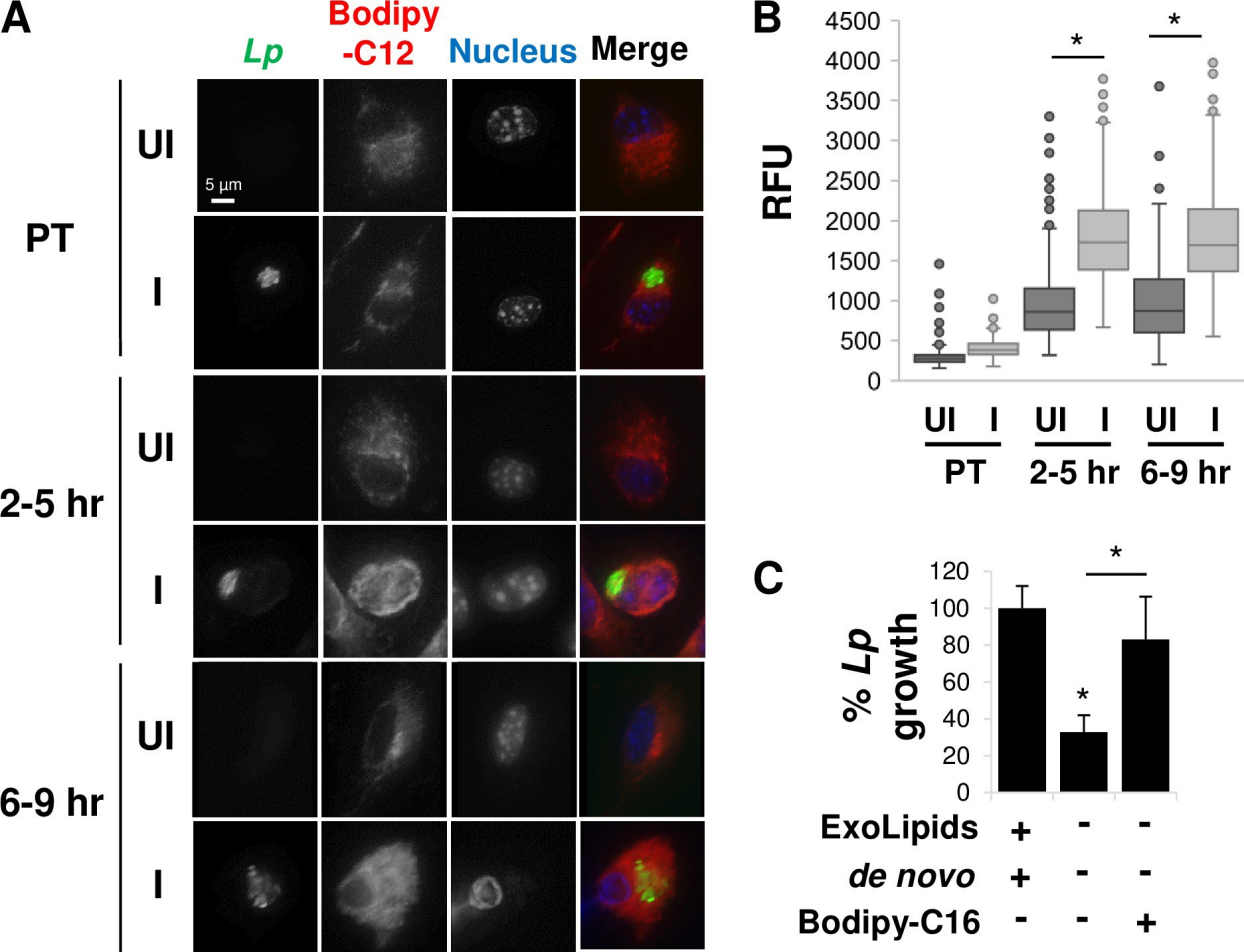
*L*. *pneumophila* infection induces lipid scavenging by host cells. **A)** Fatty acid scavenging by macrophages in response to infection. Primary A/J mouse macrophages were treated with Bodipy-C12 for 3 hrs prior to infection (pre-treated, PT) and infected with *L*. *pneumophila* (*Lp*) constitutively expressing GFP, or at 2–5 hpi or 6–9 hpi. At 17 hpi, cells were fixed, stained and visualized by microscopy. **B)** *L*. *pneumophila* infection promotes exogenous lipid uptake by host cells. The mean BODIPY-C12 signal intensity of infected (I) and uninfected (UI) cells in A) was quantified. RFU, relative fluorescence units. Data are the compilation of 3 biological replicates, each consisting of three technical replicates, analyzing at least 50 cells per technical replicate. *, One-way ANOVO Kruskal-Wallis test with a post hoc two-tailed non-parametric t test p < 0.05 after multiple correction using Mann-Whitney method. **C)** Bodipy-conjugated fatty acids are sufficient to rescue *L*. *pneumophila* intracellular growth in the absence of serum. Primary A/J mouse macrophages were infected with *L*. *pneumophila* in the presence (+) or absence (-) of exogenous lipids (ExoLipids)) or *de novo* lipid synthesis (*de novo*) by treating cells with fatostatin, in the presence or absence of Bodipy-C16 and the number of bacteria at 24 hpi was enumerated based on recovered cfus normalized to the number of bacteria in macrophages cultured in the presence of ExoLipids and *de novo* lipid synthesis at 1 hpi. Data are the mean of 3 biological replicates consisting of 3 technical replicates each. *, Student’s t-test p < 0.05 relative to untreated condition or WT cells unless otherwise indicated.

Host fatty acid availability is a key determinant of *L*. *pneumophila* pathogenesis, as the presence of fatty acids promotes *L*. *pneumophila* replication, whereas their absence or diversion to storage compartments within host cells inhibits bacterial growth (Figs 1 and 2). Since cells preferentially scavenge lipids from their environment rather than synthesize them *de novo*, we hypothesized that the fatty acid requirement for *L*. *pneumophila* replication may be satisfied by scavenging them from environmental resources. To test this idea, macrophages were treated with the fluorophore-conjugated fatty acid Bodipy-C12 at three different points during the infection cycle: i) for 3 hours prior to infection (pre-treated, PT); ii) from 2–5 hpi coinciding with vacuole remodeling and the onset of bacterial replication; or iii) 6–9 hpi during active bacterial replication. We then measured incorporation of fluorescence signal at 17 hpi comparing infected cells and uninfected cells within the same population of cells (Fig 3A). Host cells treated with Bodipy-C12 prior to infection showed no difference in signal intensity at 17 hpi compared to uninfected cells (Fig 3B). In contrast, there was significantly enhanced accumulation of Bodipy-C12 in infected cells treated at 2–5 hpi and 6–9 hpi when compared to uninfected cells (Fig 3B). Moreover, the accumulation of Bodipy-conjugated fatty acids correlated with restoration of the *L*. *pneumophila* growth defect observed in the absence of exogenous lipids (Fig 3C), indicating that *L*. *pneumophila* was able to appropriate this lipid source. Thus, *L*. *pneumophila* infection not only induces host cell acquisition of lipids from the environment but those scavenged lipids enhance *L*. *pneumophila* intracellular replication.

### Limiting host lipid availability impacts LCV membrane expansion and integrity

**Fig 4:**
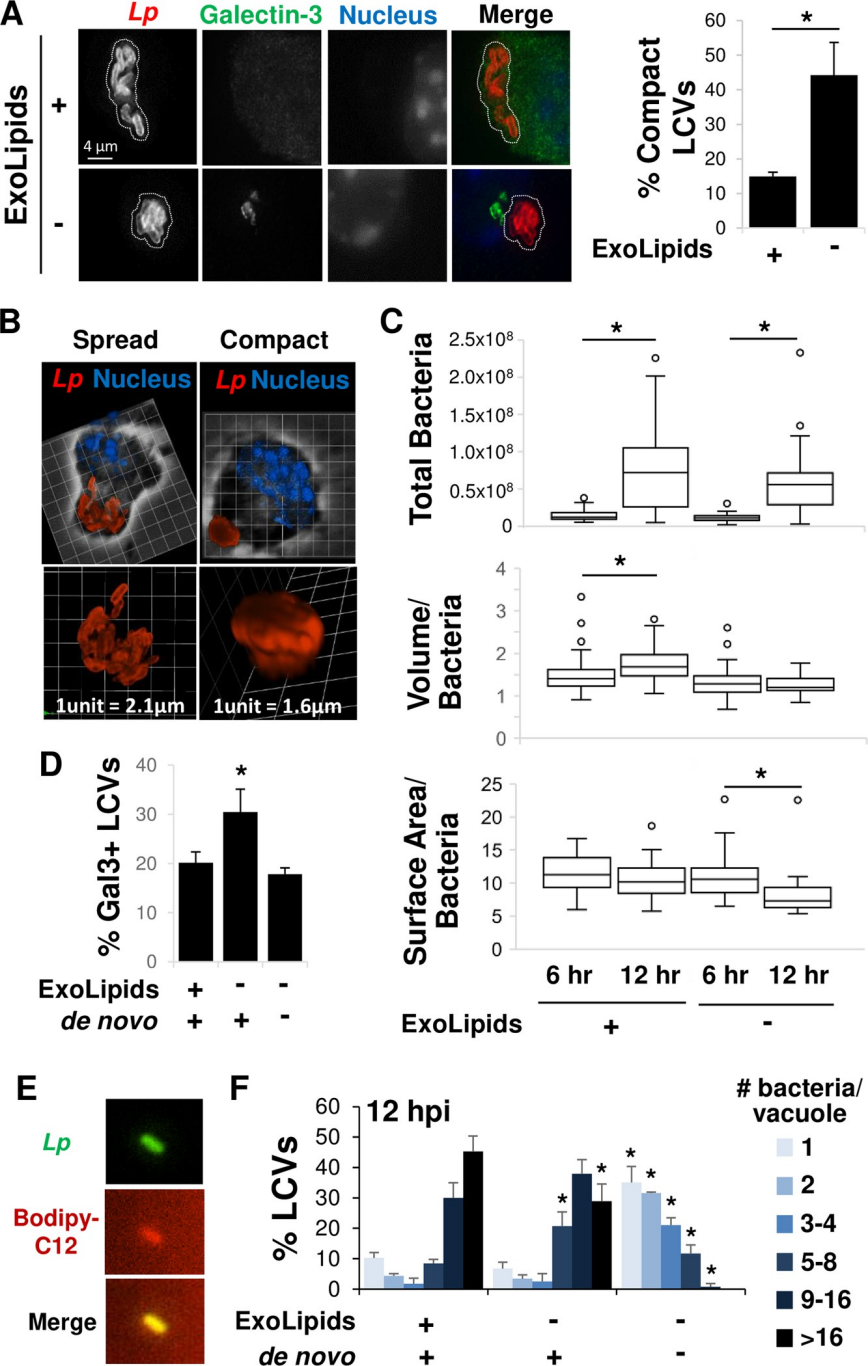
Limiting host cellular lipid availability restricts LCV membrane expansion, LCV integrity and bacterial growth. **A)** In the absence of exogenous lipids, *L*. *pneumophila* adopt a compact organization in three-dimensional space. Primary A/J mouse macrophages were infected with *L*. *pneumophila* (*Lp*) in the presence (+) or absence (-) of exogenous lipids (ExoLipids) for 12 hrs then fixed, stained and visualized by fluorescence microscopy (left panel). The number of LCVs exhibiting a compact organization were enumerated (right panel). Compact LCVs were defined as having bacteria aligned with their sidewalls juxtaposed to one another and collectively contained within a spherical shape with no bacteria extending at an angle beyond the boundary of the sphere. Additional examples of spread and compact LCVs are shown in S4 Fig. A dotted white line indicates the contour of the bacteria within the LCV. **B)** Three-dimensional deconvolution of LCVs from experiments in A) showing representative images of a spread LCV in the presence of exogenous lipids and a compact LCV in the absence of exogenous lipids. The upper panel shows the entire cell, with the darker area surrounded by white shading defining the cell boundary, while the lower panel is a magnified image of the LCV alone. The scale grid is shown in white, with the length of a single square unit indicated. In compact LCVs, bacteria can be so tightly packed together that the deconvolution software is unable to define individual bacteria. **C)** Restriction of host lipid resources diminishes LCV volume and surface area. LCV volume, based on the continuous three-dimensional space occupied by the bacteria and surface area, based on the contour of the bacteria as in B) was quantified at 6 and 12 hpi, scoring ∼50 LCVs each. *, One-way ANOVO Kruskal-Wallis test with a post hoc two-tailed non-parametric t test p < 0.05 after multiple correction using Mann-Whitney method. **D)** Reducing host lipid availability causes vacuole destabilization. Colocalization of LCVs with the host protein Galectin-3 in experiments in A) was quantified. **E)** *L*. *pneumophila* acquire fatty acids from the host cell. Primary A/J mouse macrophages were treated with Bodipy-C12 for 3 hours beginning at 6 hpi (as in Fig 3A), bacteria released from host cells at 24 hpi were harvested from culture supernatants, fixed and visualized by microscopy. **F)** Severely limiting host lipid availability impairs *L*. *pneumophila* replication. The number of bacteria per LCV in A) was quantified. A, D, F) Data are the mean ± standard deviation of 3 biological replicates consisting of 3 technical replicates each, scoring ∼50–100 LCVs per technical replicate. *, Student’s t-test p < 0.05 relative to growth in the presence of exogenous lipids and *de novo* lipogenesis.

Maintaining the LCV is critical for *Legionella* pathogenesis [8,36], requiring expansion of the LCV membrane capacity to accommodate the growing number of bacteria during replication. The temporal correlation between fatty acid scavenging by the host cell and bacterial replication (Fig 3) could be due to a role for host fatty acids in LCV membrane expansion and/or bacterial growth. To examine the effects of host lipid availability on the architecture of the LCV, macrophages were infected with bacteria and fluorescence microscopy combined with three-dimensional deconvolution was used to compare the morphology of the LCV in the presence and absence of exogenous lipids. In the presence of exogenous lipids, the bacteria were typically spread out, often aligned end-to-end and with multiple orientations in three-dimensional space (Figs 4A and 4B and S4). In the absence of exogenous lipids, the organization of bacteria was strikingly more compact, with bacteria typically bundled together with their sidewalls juxtaposed to one another (Figs 4A and 4B and S4). Thus, when lipids were abundant, LCVs typically exhibited an unstructured, amorphous shape whereas when lipids were limited, LCVs were compact, adopting a more defined, spherical shape.

To study this phenomenon in more detail, we examined the volume and surface area of the LCV in three-dimensional space (Fig 4B) between 6 hpi (when vacuole remodeling is complete and bacteria begin to grow) and 12 hpi (after several rounds of bacterial replication), in the presence and absence of exogenous lipids. Since the number of bacteria is reduced in the absence of exogenous lipids (Figs 1A and 2A and 2B), we normalized each measurement to the total bacteria within the LCV based on total fluorescence intensity (Fig 4C, upper panel), given the strong positive correlation between the number of bacteria and fluorescence signal (S5 Fig). In the presence of exogenous lipids, we observed an increase in the volume of the LCV, based on the continuous volume occupied by the bacteria (Fig 4C, middle panel), while the surface area of the LCV, based on the contour of the bacteria in three-dimensional space, remained constant (Fig 4C, lower panel) between 6 and 12 hpi. In contrast, in the absence of exogenous lipids, despite an increase in bacterial numbers (Fig 4C, upper panel), there was no concomitant increase in the volume per bacterium (Fig 4C, middle panel), and the surface area per bacterium decreased over time (Fig 4C, lower panel). Thus, comparing LCVs at 12 hpi between the two conditions, compact LCVs had both decreased volume and surface area relative to spread LCVs, and consequently, increased bacterial density (S6 Fig). Since limiting fatty acids decreased the surface area and thus, the extent of the LCV membrane, we also examined its impact on LCV integrity. To do this, fluorescence microscopy was used to inspect LCVs for co-localization with the host protein Galectin-3 (Fig 4A), an established marker for vacuole membrane damage [8,36,37]. Notably, we detected an increase in the percentage of LCVs staining positive for Galectin-3 in the absence of exogenous lipid (Fig 4D). Thus, limited host cell lipid availability during bacterial replication constrains LCV membrane expansion, resulting in altered LCV structure and increased frequency of LCV rupture.

The link between host lipid availability, LCV expansion and LCV rupture led us to predict that the severe reduction in *L*. *pneumophila* numbers observed in the absence of both exogenous lipids and *de novo* fatty acid synthesis (Fig 1A) was due to a higher percentage of LCVs undergoing destabilization. However, under this condition, the number of Galectin-3 positive LCVs was reduced to that observed when lipids were abundant (Fig 4D). One possible explanation for this was that in addition to LCV membrane expansion, host fatty acid availability also impacts *L*. *pneumophila* proliferation. In this case, reduced numbers of bacteria would require less LCV membrane, decreasing the likelihood of LCV destabilization when LCV membrane expansion is limited. Consistent with the idea that host fatty acids fuel *L*. *pneumophila* growth, we noted that bacteria isolated from macrophages supplemented with Bodipy-conjugated fatty acids were fluorescently labelled (Fig 4E), indicating the bacteria had acquired fluorescent fatty acids fed to host cells. To investigate the impact of host lipid availability on *L*. *pneumophila* replication, macrophages were infected in the presence of exogenous lipids and *de novo* lipid synthesis, the absence of exogenous lipids or both lipid resources, and the number of bacteria per LCV was determined at 12 hpi. In the presence of exogenous lipids and *de novo* lipid synthesis, most LCVs contained large numbers of bacteria, with 30% of LCV harboring 9–16 bacteria and 45% harboring >16 bacteria (Fig 4E). The absence of exogenous lipids resulted in a reduction in the percentage of LCVs with >16 bacteria and an increase in the percentage of LCVs containing 5–8 bacteria (Fig 4F), consistent with a decrease in the total bacteria isolated from infected macrophages when measured based on recovered colony forming units (Figs 1A and 2A and 2B). Even more dramatic, the absence of both exogenous lipids and *de novo* lipid synthesis led to a drastic downward shift in the number of bacteria per LCV, with the majority of vacuoles containing only 1–4 bacteria reflecting significantly reduced bacterial replication (Fig 4F). Thus, severely restricting host lipid availability impedes bacterial growth, which protects against LCV destabilization.

### Differential effects of palmitate and oleate on *L*. *pneumophila* intracellular growth are not due to toxicity towards *L*. *pneumophila*

Certain fatty acids have been shown to be toxic to bacteria at high extracellular concentrations [38]. One possible explanation for the ability of palmitate, but not oleate, to restore *L*. *pneumophila* growth when added during infection (Fig 1A) was that oleate is more toxic to *L*. *pneumophila*. To test this, we first examined *L*. *pneumophila* growth in bacteriological medium supplemented with increasing concentrations of palmitate or oleate. Concentrations up to 5 μM of either fatty acid did not cause a difference in growth when compared to bacteriological medium alone (S7A Fig). Notably, 20 μM of either fatty acid, equivalent to the concentration added to host cells for rescue experiments (Fig 1), did not affect *L*. *pneumophila* replication, although it did result in extended lag times compared to that in the absence of fatty acid supplementation (9 ± 1 hr), with a more pronounced defect in the presence of oleate (23 ± 2 hr) compared to palmitate (18 ± 2 hr) (S7A Fig). To determine if the delayed lag time was responsible for the differential impacts of oleate and palmitate on *L*. *pneumophila* numbers within host cells, we infected macrophages for 1 hour, rinsed the cells to remove non-internalized bacteria, then added palmitate or oleate to avoid any potential toxic effects of the fatty acids to *L*. *pneumophila* prior to uptake into host cells. Under these conditions, there was no difference in the onset of bacterial replication, as the percentage of LCVs containing 1, 2 or 4 bacteria between palmitate- or oleate-supplemented macrophages was similar to each other and untreated cells (S7B Fig). Thus, the differential effect of palmitate and oleate on *L*. *pneumophila* replication in host cells was not due to oleate impeding the onset of bacterial replication.

### Oleate-induced, but not palmitate-induced, lipid droplets restrict *L*. *pneumophila* access to stored lipids

**Fig 5:**
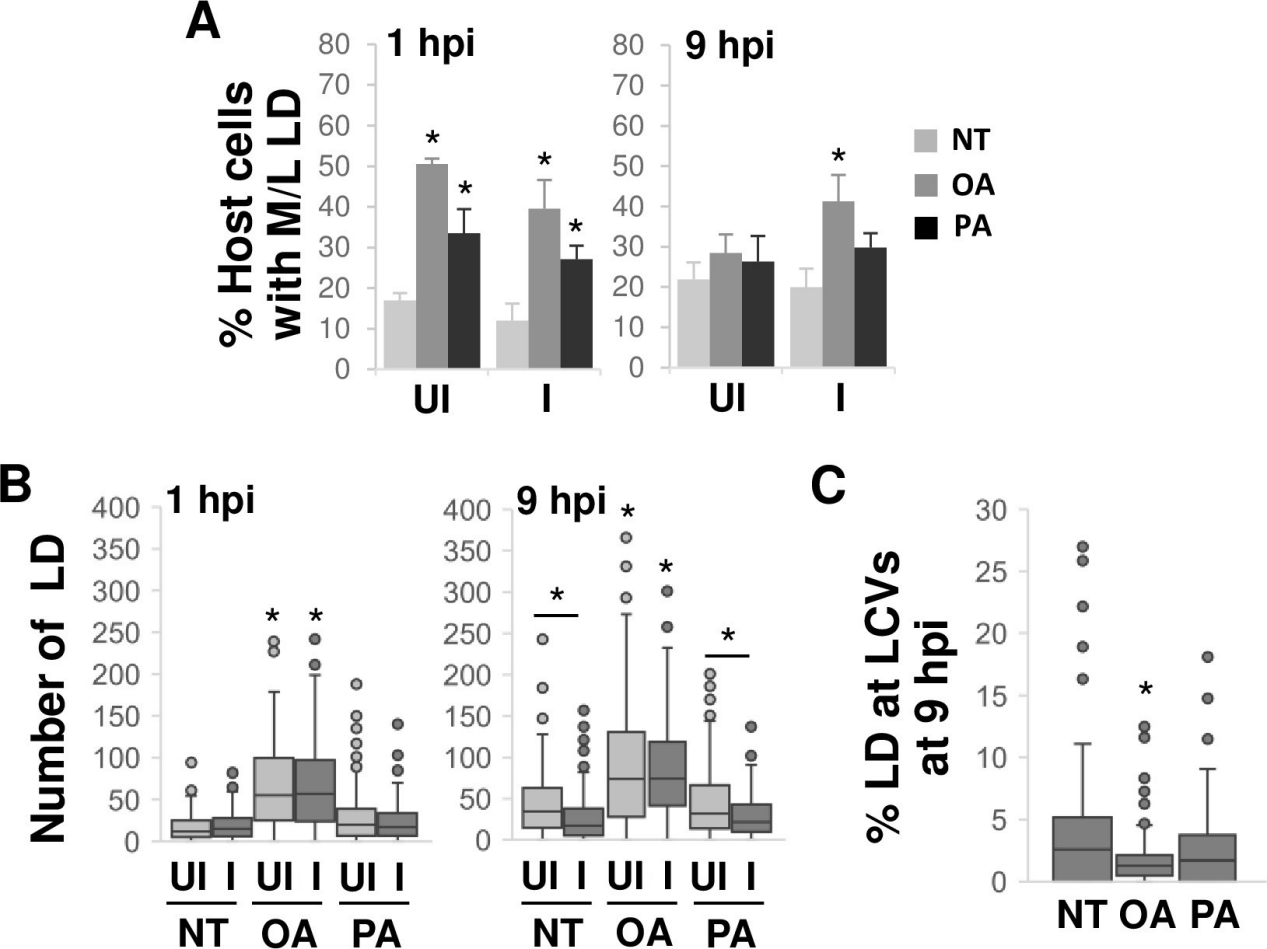
Oleate but not palmitate-mediated induction of lipid droplets restricts *L*. ***pneumophila* access to host lipids. A)** Lipid droplet size is maintained in oleate but not palmitate-treated cells during active bacterial replication. Primary bone marrow-derived A/J murine macrophages were pre-treated with oleate (OA) or palmitate (PA) for 18 hrs to induce lipid droplet formation (as in S3 Fig), rinsed and then infected with *L*. *pneumophila* (*Lp*) in the absence of exogenous lipids by culturing cells in medium lacking serum. NT, no treatment control macrophages not pre-exposed to fatty acids and cultured in the presence of serum. At 1 and 9 hpi, cells were fixed, stained and visualized by fluorescence microscopy (S7 Fig). Lipid droplet size in uninfected (UI) and infected (I) macrophages were scored (as in S3 Fig) and the percentage of cells with medium and/or large (M/L) lipid droplets (LD) were quantified. Data are the mean of 3 biological replicates scoring >200 cells in each condition at each time point, and similar numbers of infected and uninfected cells. *, Student’s t-test p < 0.05 relative to untreated condition or WT cells unless otherwise indicated. **B)** The abundance of lipid droplets in oleate, but not palmitate-treated cells, is maintained during bacterial replication. Lipid droplet numbers in uninfected (UI) and infected (I) macrophages in A) were enumerated (as in S3 Fig). **C)** Lipid droplet sequestration at the LCV is restricted in oleate but not palmitate-treated cells. The number of lipid droplets colocalizing with the LCV was quantified, normalizing to the total number of lipid droplets per cell and the number of bacteria per LCV. B-C) Data are the compilation of 2 biological replicates, scoring >200 cells in each condition at each time point, with similar numbers of infected and uninfected cells. *, One-way ANOVO Kruskal-Wallis test with a post hoc two-tailed non-parametric t test p < 0.05 after multiple correction using Mann-Whitney method.

Since stored lipids cannot rescue *L*. *pneumophila* replication defects in the absence of exogenous lipids and *de novo* lipogenesis (Fig 2A), we hypothesized inducing lipid storage could sequester host lipids from the bacteria and inhibit their growth. To test this idea, we examined the fate of lipid droplets during *L*. *pneumophila* infection. Macrophages were treated with oleate or palmitate, rinsed to remove excess fatty acids, and then challenged with *L*. *pneumophila*. At 1 hpi and 9 hpi, the size and abundance of the lipid droplets based on the staining pattern of Perilipin2 (PLIN2), a lipid droplet-specific coat protein [39] (as in S3 Fig) was compared between infected and uninfected cells within the same cell monolayer (S8 Fig). At 1 hpi, the percentage of host cells with medium and/or large lipid droplets was elevated in both oleate- and palmitate-treated cells when compared to the no treatment condition, with a similar trend observed between uninfected and infected cells (Fig 5A). At 9 hpi, the percentage of uninfected cells with medium and/or large lipid droplets for both oleate and palmitate-treated cells was similar to untreated cells (Fig 5A), demonstrating depletion of lipid droplet contents. A similar result was observed for infected cells treated with palmitate (Fig 5A). In contrast, the percentage of infected cells with medium and/or large lipid droplets remained strikingly elevated in oleate-treated cells (Fig 5A), indicating that lipids in oleate-induced lipid droplets were not mobilized during infection. A similar phenomenon was observed when comparing the abundance of lipid droplets under these conditions. At 1 hpi, the number of lipid droplets in both uninfected and infected cells was elevated in oleate-treated cells but not palmitate-treated cells when compared to untreated control cells (Fig 5B). At 9 hpi, for both the no treatment condition and palmitate-treated cells, the number of lipid droplets in infected cells was reduced compared to uninfected cells, suggesting depletion of lipid droplets in response to *L*. *pneumophila* infection (Fig 5B). In stark contrast, the number of lipid droplets in oleate-treated cells remained elevated in infected cells when compared to uninfected cells, indicating maintenance of oleate-generated lipid droplets during infection (Fig 5B). Moreover, the percentage of total lipid droplets colocalizing with the LCV in oleate-treated cells was significantly lower than in untreated cells and palmitate-treated cells (Fig 5C), suggesting differential spatial segregation of lipids droplets from LCVs between oleate and palmitate treatment conditions. Collectively, these results demonstrated distinct differences between oleate- and palmitate-generated lipid droplets. Lipids from oleate-induced lipid droplets are not available to *L*. *pneumophila* during infection, thus explaining the inhibitory effect of oleate on *L*. *pneumophila* intracellular growth (Fig 2A). Conversely, while palmitate-generated lipid droplets contents appear to be more accessible, their inability to restore *L*. *pneumophila* growth (Fig 2A) suggests pre-treating host cells with palmitate likely imposes restrictions on *L*. *pneumophila* replication through another mechanism(s).

### Inducing lipid storage impacts LCV architecture and integrity

**Fig 6:**
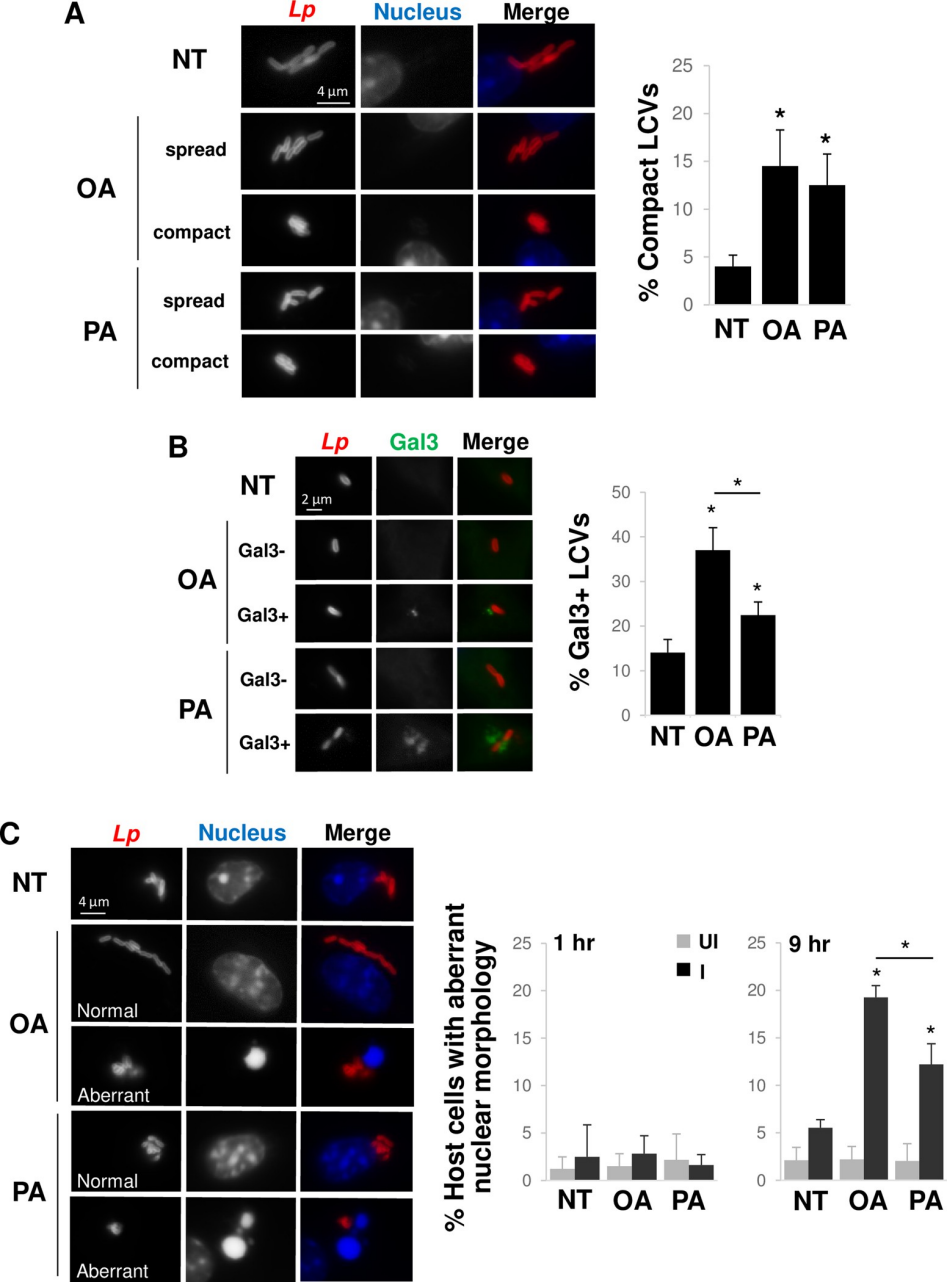
Inducing host cell lipid storage impacts LCV architecture and integrity. **A)** Inducing lipid droplet formation restricts LCV membrane expansion. Primary bone marrow-derived A/J murine macrophages were pre-treated with oleate (OA) or palmitate (PA) for 18 hrs to induce lipid droplet formation, rinsed and then infected with *L*. *pneumophila* (*Lp*) in the absence of exogenous lipids by culturing cells in medium lacking serum. NT, no treatment control macrophages not pre-exposed to fatty acids and cultured in the presence of serum. At 9 hpi, cells were fixed, stained and visualized by fluorescence microscopy (left panel). Compact LCVs were scored as described in Fig 4 and quantified (right panel). **B)** Treating cells with oleate and palmitate leads to LCV rupture. Primary bone marrow-derived A/J mouse macrophages were infected with *L*. *pneumophila* in the presence of oleate or palmitate for 6 hours, then fixed, stained and visualized by fluorescence microscopy (left panel). Colocalization of LCVs with Galectin-3 (Gal3) was scored (right panel). **C)** Inducing lipid droplet formation leads to host cell death during infection. Primary bone marrow-derived A/J murine macrophages were pre-treated with oleate or palmitate then challenged with *L*. *pneumophila* as in A). Cells were fixed, stained and visualized by fluorescence microscopy (left panel), and uninfected (UI) and infected (I) cells were scored for aberrant nuclear morphology at 1 and 9 hpi (right panel). A-C) Data are the mean ± standard deviation of 3 biological replicates consisting of 3 technical replicates each, scoring ∼50–100 LCVs per technical replicate (A,B) or ∼50–100 LCVs for uninfected and infected cells per technical replicate (C). *, Student’s t-test p < 0.05 relative to NT control cells (A,B) or fatty acid-treated cells to NT in uninfected and infected, respectively (C), unless otherwise indicated.

While examining the effect of lipid storage on *L*. *pneumophila* replication, we speculated that oleate and palmitate might impact LCV expansion and stability. Interestingly, pre-treating host cells with either oleate or palmitate resulted in an increase in the number of LCVs with a compact organization of bacteria (Fig 6A), indicating limited LCV membrane expansion, similar to the effect observed when limiting exogenous lipids (Fig 4). Moreover, oleate supplementation resulted in a larger percentage of LCVs staining positive for Galectin-3 (Fig 6B), indicating LCV rupture. Palmitate treatment had a similar effect, although less severe than oleate (Fig 6B), indicating oleate is more disruptive to LCV integrity. Since LCV rupture often leads to host cell death [8,40], we examined whether the effect of fatty treatment on host cells rendered them less likely to survive infection. At 1 hpi, the majority of host cells, irrespective of infection, were not undergoing programmed cell death, as detected by aberrant nuclear morphology (Fig 6C). However, similar to the results for LCV rupture, a higher number of oleate- and palmitate-treated cells showed signs of cell death when compared to uninfected cells at 9 hpi (Fig 6C), with the effect being more severe for oleate-treated cells (Fig 6C). Thus, fatty acid-stimulated production of lipid droplets has multiple effects on *L*. *pneumophila* intracellular replication, including limiting LCV membrane expansion, inducing LCV destabilization and increasing host cell death.

## Discussion

Here, we demonstrate a critical role for host fatty acids in promoting both *L*. *pneumophila* vacuole expansion and bacterial replication. When host cell lipids are abundant, bacteria grow optimally and spread out in three-dimensional space, unencumbered by LCV membrane expansion (Fig 4). However, when host lipid resources become limited, for example through the lack of exogenous lipids (Fig 1) or diverting fatty acids for storage in lipid droplets (Figs 2 and 5), *L*. *pneumophila* compensate by minimizing LCV expansion to support their replication, which leads to compact LCVs (Figs 4 and 6). The limited expansion of the LCV membrane sometimes leads to LCV destabilization (Figs 4 and 6), further restricting bacterial load in the cell. When host lipids become scarce, *L*. *pneumophila* replication is grossly impaired, resulting in severely diminished numbers of bacteria within host cells (Figs 1, 2 and 4). Thus, fatty acid availability regulates the extent of both LCV expansion and bacterial replication, establishing host fatty acid resources as a critical determinant of bacterial burden in macrophages and thus, *L*. *pneumophila* pathogenesis.

Fatty acids contribute to *L*. *pneumophila* intracellular replication in multiple ways. LCV membrane expansion requires fatty acid-containing phospholipids and sphingolipids, which serve as main constituents of eukaryotic membranes. Acquisition of lipids could occur through their direct insertion at membrane contact sites [41,42] or fusion of vesicles with the LCV membrane [15–18,20,21]. Moreover, since the structure and function of membranes can be greatly impacted by their composition, such as the nature of the phospholipid and sphingolipid acyl chains, the types of fatty acids available are also likely to affect LCV architecture and stability, either directly, by altering the pool of lipids available for incorporation into the LCV membrane or indirectly, by affecting cellular processes involving membranes, such as vesicle trafficking or tubular ER rearrangements, that are important for LCV integrity [15–21,43,44]. In addition to LCV expansion, *L*. *pneumophila* also utilize fatty acids and/or complex lipids for intracellular growth, as demonstrated by fluorescent labelling of bacteria when infected macrophages were fed fluorophore-conjugated fatty acids (Fig 4E) and, in the amoebae *Dictyostelium discoideum*, incorporation of ^13^C into *L*. *pneumophila* when infected amoeba were supplemented with [^13^C]palmitate [45]. Additionally, Hilbi and colleagues demonstrated that a *L*. *pneumophila* mutant lacking a homolog of *fadL*, which encodes a fatty acid import protein, accumulated less ^13^C-containing molecules and grew more poorly in *D*. *discoideum* [45]. Finally, *Legionella gormanii* membranes were recently shown to surprisingly contain the sphingolipid ceramide, implicating host-derived fatty acid-containing molecules in performing a structural role in the bacterium as well [46].

Despite the contribution of fatty acids to two seemingly separate processes, LCV membrane expansion and *L*. *pneumophila* replication are likely to be intimately linked. In the simplest model, LCV membrane expansion would directly correlate with *L*. *pneumophila* growth. However, under normal conditions, the bacteria adopt a spread configuration (Figs 4 and S4), with lower bacterial density within the LCV (S6 Fig). It is presently unknown why or how *Legionella* replicates in this manner. While the shape of the LCV is likely due, at least in part, to the physical constraints of the surrounding space (such as other organelles), the spread configuration may also benefit the bacteria under certain conditions. For example, the increase volume could provide additional space for the bacteria to grow and divide whereas the increased surface area could allow for enhanced uptake of nutrients from the cytoplasm, facilitate vesicle docking and fusion for LCV membrane expansion, and/or allow for optimal effector translocation, which requires the Dot/Icm secretion system be localized to the bacterial poles [47,48]. When host cell lipid resources are limited, LCV membrane expansion is restricted in favor of *L*. *pneumophila* replication (Fig 4). While this adaptation results in a more compact LCV containing a higher density of bacteria (S6 Fig), it allows for optimal bacterial growth across a gradient of available lipid resources. Nevertheless, *L*. *pneumophila* presumably does not normally employ a compact LCV when lipid resources are abundant, as it can lead to catastrophic LCV destabilization (Fig 4).

Since adequate fatty acid resources are critical for *L*. *pneumophila* intracellular replication, control over fatty acid resources provides an opportunity for both the pathogen and host to dictate the outcome of infection. For example, host cell fatty acid scavenging induced during infection (Fig 3) may be mediated by *L*. *pneumophila* through the activity of secreted effectors, ensuring sufficient intracellular lipids pools to support robust LCV expansion and bacterial growth. Additionally, *L*. *pneumophila* appear to control the balance between lipid production and storage, as two *L*. *pneumophila* effectors, LegK7 and LegA3/AnkH that promote LCV integrity [36] regulate host lipid metabolism, specifically shifting lipid storage to lipid mobilization and synthesis [49,50]. LegK7 modulates the host Hippo pathway, which includes members of the PPARγ gene regulon [49], whereas LegA3/AnkH, indirectly regulates Hippo signaling by manipulating host cell transcription [50]. Although the deletion of *legK7* and/or *legA3/ankH* did not affect *L*. *pneumophila* growth in the absence of *de novo* lipogenesis (S9 Fig), it would not be surprising if other *L*. *pneumophila* effectors similarly function to modulate host lipid metabolic pathways [51]. While *L*. *pneumophila* attempts to increase fatty acid availability, fatty acid restriction provides the host cell with a means to defend against *L*. *pneumophila*. Consistent with this idea, diverting host cell fatty acids to storage compartments, either through the activation of PPARγ or the induction of lipid droplet formation impairs, *L*. *pneumophila* replication (Fig 2). Since oleate-induced lipid droplets had decreased colocalization with the LCV and were not depleted from the host cell during active bacterial replication (Fig 5), their induction appears to be a particularly effective method for segregating lipids from *L*. *pneumophila* during infection. Thus, it is unlikely that oleate-induced lipid droplets play a direct role in cytotoxic killing of bacteria via a mechanism involving fusion of lipid droplets with bacteria-containing vacuoles [52,53], although this cannot be ruled out. Instead, the inhibitory effect of oleate appears to impact the LCV itself, by restricting LCV expansion and increasing LCV rupture (Fig 6). Oddly, the *L*. *pneumophila* effector LegA15 has been reported to stabilize lipid droplets in immortalized murine macrophages [54], although it is not clear how this would benefit the bacteria, particularly given the minimal *L*. *pneumophila* replication observed under these conditions [54].

In contrast to oleate-induced lipid droplets, palmitate-induced lipid droplets were more likely to associate with LCVs and were consumed during infection of macrophages (Fig 5), and have been shown to colocalize with the LCV in *D*. *discoideum* [45], a process mediated by the *L*. *pneumophila* effector LegG1 [45]. However, while palmitate-induced lipid droplets enhance *L*. *pneumophila* replication in *D*. *discoideum* [45], this did not occur in macrophages (Fig 2A), although they were consumed in both host cell types. The difference may be due to the effects of palmitate on the mammalian immune system, which includes the induction of M1 polarization, activation of TLR signaling and production of proinflammatory cytokines [55–57]. These host cell responses may be responsible for the failure of palmitate treatment prior to infection to restore *L*. *pneumophila* growth (Fig 2A), and instead leads to defects in LCV expansion and integrity (Fig 6). Similarly, oleate-mediated effects on immunity, including driving M2 polarization by impacting IL-4 induced gene expression and activating PPARγ [57,58] may also contribute to its impact on *L*. *pneumophila* replication in macrophages (Fig 2C) [59–64]. Thus, the role of host cell fatty acids in *L*. *pneumophila* pathogenesis is likely to be complex, with the types of fatty acids and their dependent cellular processes impacting the outcome of infection.

Collectively, our data demonstrate that *L*. *pneumophila* exploit multiple host lipid resources for both LCV expansion and bacterial growth. However, acquisition of different cellular lipid pools is complicated as not all are freely accessible to the bacterium. As a consequence, the relative distribution of fatty acids to different cellular pools and metabolic flux between them have profound effects on *L*. *pneumophila* virulence. When host lipid resources are restricted, *L*. *pneumophila* appears to modulate its intracellular replication to growth within compact LCVs, thereby optimizing lipid availability for bacterial growth. However, this strategy imposes constraints on LCV expansion, which can lead to defects in LCV integrity. Thus, fatty acids availability and control over their metabolic fates are key determinants in both the maintenance of a replication compartment and growth of an intracellular pathogen, adding another dimension to fatty acid-mediated metabolic immunity [65–70] that is likely to have broad implications in microbial pathogenesis.

## Materials and methods

### Bacterial strains, cultured cells and growth media

*Legionella pneumophila* Philadelphia-1 strain Lp02 (wild type) and Lp03 (a Dot/Icm translocation deficient (*dot*-) and thus, avirulent strain) [71] were cultured at 37°C in liquid *N*-(2-acetamido)-2-aminoethanesulfonic acid (ACES) buffered yeast extract (AYE) medium or on solid charcoal ACES-buffered yeast extract (CYE) medium [72] containing 0.4 mg/ml L-cysteine sulfate (Sigma) and 0.135 mg/ml ferric nitrate (Sigma) and, when appropriate, 0.1 mg/ml thymidine (Sigma) (CYET). For host cell lipid scavenging experiments, cells were infected with *L*. *pneumophila* strain TO1476, a thymidine auxotroph revertant of Lp02 [73] harboring p*P*_*pacS*_::*egfp* [74] constitutively expressing the enhanced green fluorescent protein (GFP). Primary bone marrow-derived macrophages from A/J and C57B/L6 (WT and *Fasn*-/-) mice were isolated as previously described [71]. Primary macrophages and wild type and PPARγ-/- immortalized primary murine macrophages derived from C57BL/6 mice [35] were cultured in RPMI medium containing 10% fetal bovine serum (FBS) at 37°C in the presence of 5% CO_2_. Macrophages isolated from C57BL/6 mice were infected with a *L*. *pneumophila flaA* deletion strain (a kind gift from Dr. Michele Swanson, University of Michigan) and medium was supplemented with 40 ng/ml of Macrophage Colony-Stimulating Factor (M-CSF) (Peprotech).

### Intracellular growth assays

Growth of *L*. *pneumophila* in primary bone marrow-derived murine macrophages was performed as previously described [71]. Briefly, *L*. *pneumophila* were grown to post-exponential phase (absorbance at 600 nm, A_600_ = 3.8–4.0, and judging motility as determined by visual inspection using an inverted microscope equipped with a 40x lens) and used to challenge 1x10^5^ macrophages at a MOI = 1 for 1 hr. Cells were rinsed three times with culture medium and at 1 and 24 hours, cells were lysed with 0.05% saponin at 37°C for 10 min and diluted lysates were plated on bacteriological medium for colony forming units (cfus). For testing exogenous lipids, infected cells were rinsed three times with medium lacking serum at 1 hpi and maintained in medium lacking serum through 24 hrs. For inhibitors, cells were treated with 4 μM fatostatin (Sigma), 2 μM A939572 (Sigma), 100 μM myriocin (Sigma) or 10 μM rosiglitazone (Sigma), subsequent to rinsing at 1 hpi. For palmitate and oleate complementation experiments, cells were treated with fatty acid free (FAF) albumin (Sigma) or palmitate (Sigma) or oleate (Sigma) combined at a 1:4 molar ratio with FAF albumin in culture medium immediately prior to use and added to cells at a final concentration of 5 μM FAF albumin and 20 μM fatty acid. Untreated cells were incubated in medium containing 5 μM FAF albumin alone. For complementation with Bodipy-conjugated fatty acids, macrophage culture medium was supplemented with 1 μM BODIPY 558/568 C_16_ (Thermo Fisher Scientific) subsequent to rinsing cells at 1 hpi and maintained for the duration of the infection to 24 hrs.

### Host cell viability assays

For host viability assays, macrophages were treated as above for 24 hours (in the absence of infection) and cell death was measured based on lactate dehydrogenase release using the Cytox96 Cytotoxicity Assay (Promega) or cellular redox potential using the XTT Cell Viability and Proliferation Assay (Sigma), following the manufacturer’s instructions.

### *de novo* lipogenesis assays

*de novo* lipogenesis was assayed in primary bone marrow-derived A/J mouse macrophages, as previously described [75]. Briefly, 2.5x10^6^ cells were pre-treated for 24 hrs in RPMI in the presence of 10% FBS, in the absence of FBS, or in the absence of FBS but supplemented with 5 μM FAF and 20 μM palmitic acid or oleic acid. Media was then replace with fresh medium containing 0.5 μCi/mL [^3^H]acetate (New England Nuclear) and incubated for 6 hours. Cells were washed with 1x phosphate buffered saline (PBS). Total lipids were extracted by the method of Folch *et al*. [76] by treating cells with 700 μl of 2:1 chloroform:methanol and 450 μl 4 mM magnesium chloride. Samples were vortexed and centrifuged at 18,120 x *g* for 1 min at 25°C. The organic phase was combined with 450 μl of 2:1 CHCl_3_:MeOH and 300 μl of MgCl_2_, vortexed, and centrifuged as above. Equal volumes of the organic phase were counted by liquid scintillation. Background radioactivity from wells containing no cells but the corresponding medium was subtracted from the cellular counts and normalized to total cellular protein harvested from 2.5x10^6^ cells treated as above but that did not receive radiolabeled substrate. For protein extraction, cells were washed with 1xPBS and lysed on ice in Lysis buffer [50 mM Tris–HCl at pH 7.4, 150 mM NaCl, 1 mM EDTA, 1% Triton X-100, and 0.25% deoxycholate] containing Roche PhosSTOP phosphatase inhibitor (Millipore Sigma) and protease inhibitor cocktail (Millipore Sigma). Lysates were cleared by centrifugation at 13,300 x *g* at 4°C for 20 minutes. Total protein content was quantified using the Pierce BCA assay (Thermo Fisher Scientific) following the manufacturer’s instructions.

### *in vitro* growth assays

*L*. *pneumophila* were sub-cultured to a concentration of 0.3x10^9^ bacteria/ml (A_600_ = 0.3) in AYE medium supplemented with varying concentrations of palmitate or oleate, or 90% ethanol vehicle control and incubated at 37°C with shaking. Growth was monitored by measuring A_600_ at regular intervals. The lag phase was defined as the time at which the A_600_ increased relative to the initial inoculum. The doubling time (d_t_) was determined as follows: d_t_ = t/n where t = time of exponential phase growth (t_2_-t_1_, where t_1_ = time at the beginning of exponential growth and t_2_ = time at the end of exponential growth) and n = (logA_2_-logA_1_)/log2, where A_1_ and A_2_ = A_600_ of the bacterial culture at t_1_ and t_2_, respectively.

### Infectious center assays

To examine LCV architecture and integrity, and enumerate the number of bacteria per LCV, 4x10^5^ macrophages were plated on coverslips in a 24-well tissue culture plate and incubated overnight. *L*. *pneumophila* were grown to post-exponential phase then used to challenge macrophages at a MOI = 1 for 1 hr. Cells were rinsed 3 times with culture medium either containing FBS (exogenous lipids) or lacking FBS (no exogenous lipids). At 6 and 12 hpi, cells were fixed in 4% paraformaldehyde in 1x PBS for 30 min at room temperature in the dark. Cells were permeabilized with ice-cold methanol and stained with a chicken α-*Legionella* antibody (1:5,000) [48] and rat α-Galectin-3 antibody (Santa Cruz) (1:1,000) in 1x PBS containing 4% goat serum (Gibco) followed by goat α-chicken IgG Alexa Fluor 594 (1:500) (Molecular Probes) and goat α-rat IgG Alexa Fluor 488 (1:1,000) (Molecular Probes). Samples were mounted in SlowFade Gold Antifade reagent (Invitrogen) and imaged with a Nikon Eclipse Ti-E inverted fluorescence microscope in three dimensions acquiring Z stacks at 0.2 μm intervals using NIS Elements software (vAR 5.21.03). Images were deconvoluted and signal intensity, volume and surface area in three-dimensional space were quantified using Volocity (v7.0.0).

### Immunofluorescence microscopy

For host cell fatty acid scavenging experiments, 4x10^5^ macrophages were plated on coverslips in a 24-well tissue culture plate and incubated overnight. For pre-treatment, cell culture medium was replaced with medium containing 1 μM BODIPY 558/568 C_12_ (Bodipy-C12) (Thermo Fisher) 3 hrs prior to infection and rinsed 3 times with culture medium lacking Bodipy-C12 immediately before infection. *L*. *pneumophila* were grown to post-exponential phase then used to challenge macrophages at a MOI = 1 for 1 hr. Cells were rinsed 3 times with culture medium. For Bodipy-C12 treatment during infection, Bodipy-C12 was added to cells to a final concentration of 1 μM at 2 or 6 hpi, incubated for 3 hours, then rinsed 3 times with medium lacking Bodipy-C12. For each treatment, at 17 hpi, cells were fixed in 4% paraformaldehyde in 1x PBS for 30 min at 25°C in the dark. Cells were stained with 5 ug/ml Hoechst in PBS at 25°C for 30 min in the dark and mounted in SlowFade Gold Antifade reagent (Invitrogen) and imaged on a Nikon Eclipse Ti-E inverted fluorescence microscope in three dimensions as described above. Images were deconvoluted and signal intensity in three-dimensional space were quantified using Volocity (v7.0.0) comparing infected and uninfected cells in the same monolayer. For imaging bacteria, host cell supernatants at 24 hpi were harvested, centrifuged at 23 x *g* for 5 min to remove cell debris, then 13,300 x *g* for 2 min to isolate bacteria. Bacteria were fixed in ice-cold methanol, resuspended in 1xPBS, mounted on poly-lysine coated glass slides and visualized by microscopy.

For experiments to examine the fate of lipid droplets during infection, 2x10^5^ macrophages were plated and incubated for 5–6 hrs. FAF albumin and palmitate or oleate were combined at 1:6 molar ratio and added to cells at a final concentration of 5 μM FAF albumin and 30 μM fatty acid, and incubated for 18 hrs. Untreated cells were incubated in medium alone. Cells were then rinsed with medium to remove exogenous lipids and challenged with *L*. *pneumophila* at a MOI = 1 for 1 hour. At 1 hpi, cells were rinsed to remove non-internalized bacteria, and medium was replaced with either medium containing FBS (for no treatment control cells) or medium lacking FBS (no exogenous lipids) (for fatty acid treated cells). At 1 and 9 hpi, cells were fixed in 4% paraformaldehyde in 1x PBS for 30 min at 25°C in the dark. Cells were then permeabilized with ice-cold methanol and stained with a rabbit α-Perilipin 2 antibody (1:200) (Proteintech) and chicken α-*Legionella* antibody (1:5,000) in 1x PBS containing 4% goat serum (Gibco) followed by goat α-rabbit IgG Alexa Fluor 488 (1:1,00) and goat α-chicken IgG Alexa Fluor 594 (1:500) (Molecular Probes). Cells were stained with 5 ug/ml Hoechst in 1xPBS at 25°C for 30 min in the dark and mounted in SlowFade Gold Antifade reagent (Invitrogen). Samples were imaged on a Nikon Eclipse Ti-E inverted fluorescence microscope in three dimensions as described above.

To examine vacuole integrity, 2x10^5^ macrophages were challenged with *L*. *pneumophila* at a MOI = 1 for 1 hour. Cells were then rinsed with medium containing FBS (no treatment) or medium lacking FBS and replaced with medium containing FBS, or medium lacking FBS but supplemented with 5 μM FAF and 20 μM oleate or palmitate. At 1 and 6 hours, cells were fixed in 4% paraformaldehyde in 1x PBS for 30 min at 25°C in the dark. Cells were then permeabilized, stained for *Legionella*, Galectin-3 and host cell nuclei and imaged in three dimensions as described above.

## Supporting information

S1 Fig Palmitate and oleate similarly modulate host cell *de novo* lipogenesis. Since *de novo* fatty acid synthesis is regulated through SREBP in response to available lipids, one possible explanation for the differential growth of *L*. *pneumophila* in macrophages treated with oleate versus palmitate (Fig 1) was that oleate reduced fatty acid biosynthesis to a greater extent than palmitate, thereby having more pronounced effects on cellular fatty acid levels in the absence of exogenous lipid resources. To test this, FASN levels and [^3^H]acetate incorporation into lipids was examined under these conditions as a measure of *de novo* lipogenesis. **A)** Fatty acid treatment does not alter endogenous fatty acid synthase levels in macrophages. Primary A/J mouse macrophages were incubated in the presence (+) or absence (-) of serum (exogenous lipids, ExoLipids) or in medium lacking serum supplemented with either oleate or palmitate for 18 hrs. Cells lysates were then analyzed for FASN by Western analysis (top panel) and quantified (bottom panel), normalizing to total protein. **B)** Oleate and palmitate treatment similarly reduces *de novo* lipogenesis. Macrophage as in A) were exposed to [^3^H]acetate. Lipids were then extracted and examined for radiolabel incorporation, normalizing to total protein. The absence of exogenous lipids resulted in a 5-fold increase in the amount of lipid synthesis when compared to cells cultured in the presence of exogenous lipids, consistent with the response of host cells to activate lipogenesis when exogenous lipids are not available. When either palmitate or oleate was added, [^3^H]acetate incorporation was significantly reduced, consistent with repression of lipid biosynthetic pathways through SREBP by exogenously available fatty acids. The extent of the effect observed was similar between palmitate and oleate, indicating the differential effects of oleate and palmitate on *L*. *pneumophila* replication was not due oleate more extensively limiting *de novo* fatty acid synthesis in the absence of exogenous lipids. A-B) Data are the mean ± standard deviation of 3 biological replicates, consisting of 1–2 technical replicates each. *, Student’s t test p <0.05, relative to the cells culturing in the presence of exogenous lipids unless otherwise indicated.
(TIF)

S2 Fig Conditions of host lipid restriction do not impact host cell viability. **A)** Primary macrophages were cultured in medium in the presence (+) or absence (-) of exogenous lipids (ExoLipids) supplemented with 4 μM fatostatin, 5 μM FAF albumin plus 20 μM palmitate or oleate, 2 μM A939572 or 100 μM myriocin for 24 hours and cell death based on lactate dehydrogenase (LDH) release was determined. **B)** Primary macrophages were treated as in A) and cellular respiration based on redox potential was measured. A-B) Data are the mean ± SD of 3 biological replicates, consisting of 2 technical replicates each.
(TIF)

S3 Fig Fatty acid-induced lipid droplet formation in primary A/J mouse macrophages. **A)** Differential induction of lipid droplet production in response to fatty acid treatment. Primary bone marrow-derived A/J murine macrophages were pre-treated with oleate or palmitate for 18 hrs. Cells were rinsed, fixed, stained for the lipid droplet (LD)-specific coat protein Adipose differentiation-related protein/Perilipin2 (PLIN2) [39] and visualized by fluorescence microscopy. Macrophages generally exhibited four patterns of lipid droplets: cells with no detectable lipid droplets, based on a lack of PLIN2 puncta, or cells harboring small-, medium- or large-sized lipid droplets, based on the relative diameter of the PLIN2 puncta, with some cells containing combinations of small and medium or medium and large lipid droplets. **B)** Pre-treating macrophages with fatty acids induced lipid droplet formation compared to no treatment (NT) control cells lacking fatty acid supplementation. **C)** Pre-treating cells with oleate or palmitate resulted in a higher percentage of cells harboring medium and/or large lipid droplets compared to untreated macrophages, with a more pronounced increase for oleate-treated cells relative to palmitate-treated cells. Cells in B) were scored based on the size of the PLIN2 puncta, and the percentage of cells containing no PLIN2 puncta, small or a combination of small and medium puncta (S/M), or medium and/or large puncta (M/L) was determined. Data are the mean ± standard deviation of 3 biological replicates, analyzing >100 cells per replicate. *, Student’s t-test p < 0.05 relative to NT control cells unless otherwise indicated. **D)** Pre-treating cells with oleate, but not palmitate, increased the number of lipid droplets, particularly in macrophages harboring medium and/or large lipid droplets. Lipid droplets based on the number of PLIN2 puncta in cells in B) were enumerated and cells containing small puncta (S) or medium and/or large puncta (M/L), were compared. Data are the mean ± standard deviation of 2 biological replicates, scoring >150 cells per replicate. *, one-way ANOVO Kruskal-Wallis test with a post hoc two-tailed non-parametric t test p < 0.05 after Mann-Whitney correction for multiple testing, relative to the NT control unless otherwise indicated.
(TIF)

S4 Fig Limiting exogenous lipids leads to compact LCV architecture. In the absence of exogenous lipids, *L*. *pneumophila* adopt a compact organization in three-dimensional space. Primary A/J mouse macrophages were infected with *L*. *pneumophila* in the presence (+) or absence (-) of serum (exogenous lipids, ExoLipids) for 6 or 12 hrs, fixed, stained and visualized by fluorescence microscopy. The organization of bacteria in the LCV in three-dimensional space was analyzed by three-dimensional deconvolution. Representative LCVs at 6 and 12 hpi from experiments in Fig 4 are shown, highlighting the typical spread organization of the LCVs when lipids are abundant (+ ExoLipids) and the combination of compact and spread LCVs observed when lipids resources are limited (- ExoLipids).
(TIF)

S5 Fig Correlation between fluorescence signal intensity of bacteria and bacteria numbers per LCV. Primary A/J mouse macrophages were infected with *L*. *pneumophila* (*Lp*) for 12 hr. Cells were then fixed, stained and visualized by fluorescence microscopy. The fluorescence signal intensity of bacteria after immunostaining was compared to the number of bacteria counted within the LCV. RFU, relative fluorescence units.
(TIF)

S6 Fig Host cell fatty acids availability determines LCV expansion and structure. **A)** When host cell fatty acids resources are plentiful, the abundance of availability fatty acids to fuel LCV membrane expansion allows the bacteria spread out, adopting a spatially disordered configuration with multiple orientations in three-dimensional space. As the bacteria replicate, the volume of the LCV per bacterium increases, while the surface area required to encompass the bacteria and the space between them remains relatively constant. **B)** When host lipid resources become limited, LCV membrane expansion is constrained, causing bacteria to bundle together, aligning along their lengths with similar orientations in three-dimensional space. In this case, as the bacteria replicate, their close packing limits luminal space between them such that the volume of the LCV per bacteria remains constant, while adopting a more spherical shape minimizes the amount of LCV membrane required to encompass the bacteria, and thus a reduction in the surface area. The resulting compact structure maximizes volume per surface area, and thus the capacity of the LCV, allowing the maximum number of bacteria to be accommodated with the minimal amount of LCV membrane. As a consequence, compact LCVs are smaller in size but have a higher bacterial density than spread LCVs.
(TIF)

S7 Fig Oleate or palmitate treatment do not impact the onset of bacterial replication within host cells. **A)** Exposure of *L*. *pneumophila* to fatty acids *in vitro* results in an extended lag phase but does not impact bacterial growth. *L*. *pneumophila* were cultured in bacteriological medium lacking fatty acids supplementation (no FA) or containing increasing amounts of either oleate or palmitate, and bacterial growth based on absorbance (A) at 600 nm (A_600_) was measured over time. Plotted data are representative of 3 biological replicates, which consisted of 2 technical replicates each. Reported lag phase and doubling times are the mean ± standard deviation of the 3 biological replicates represented by the data plot. *, Student’s t test p <0.05, relative to cells cultured in medium lacking fatty acid supplementation. #, Student’s t test p <0.05, relative to cells cultured in medium supplemented with the same concentration of palmitate. **B)** Fatty acid treatment of host cells does not impact the onset of bacterial replication during infection. Primary A/J mouse macrophages were challenged with *L*. *pneumophila* for 1 hour, rinsed and cultured in medium supplemented with palmitate or oleate and compared to cells culture in medium alone (NT, no treatment). At 6 hpi, cells were fixed, stained and visualized by fluorescence microscopy, enumerating the number of bacteria per LCV. Data are the mean ± standard deviation of 3 biological replicates, scoring >100 vacuoles per replicate.
(TIF)

S8 Fig Fate of oleate and palmitate-induced lipid droplets in *L*. *pneumophila*-infected macrophages. **A)** Primary bone marrow-derived A/J murine macrophages were pre-treated with oleate (OA) or palmitate (PA) for 18 hrs (as in S3 Fig), rinsed and then infected with *L*. *pneumophila* in the absence of exogenous lipids by culturing cells in medium lacking serum. NT, no treatment control macrophages not pre-exposed to fatty acids and cultured in the presence of serum. At 1 and 9 hpi, cells were fixed, stained for *L*. *pneumophila* (*Lp*), the lipid droplet coat protein Perilipin2 (PLIN2) and nuclei and visualized by fluorescence microscopy.
(TIF)

S9 Fig Loss of LegA3/AnkH and/or LegK7 does not attenuate *L*. *pneumophila* growth in macrophages impaired for *de novo* lipogenesis. Primary bone marrow-derived A/J mouse macrophages were infected with the indicated *L*. *pneumophila* strains in the presence (+) or absence (-) of *de novo* lipid synthesis by treating cells with the SREBP1 inhibitor fatostatin. Bacterial growth was quantified based on recovered colony forming units (cfus) from host cell lysates at 24 hrs, and normalized to wild type (WT) bacteria in untreated host cells by the number of intracellular bacteria at 1 hpi. Data are the mean ± standard deviation of 3 biological replicates consisting of 3 technical replications each.
(TIF)
