# Supplementary figures and images for: Legionella pneumophila usurps host cell lipids for vacuole expansion and bacterial growth
Source: PLoS Pathog. 2024 Feb 22;20(2):e1011996. doi: 10.1371/journal.ppat.1011996 (PMC10883544; doi:10.1371/journal.ppat.1011996)

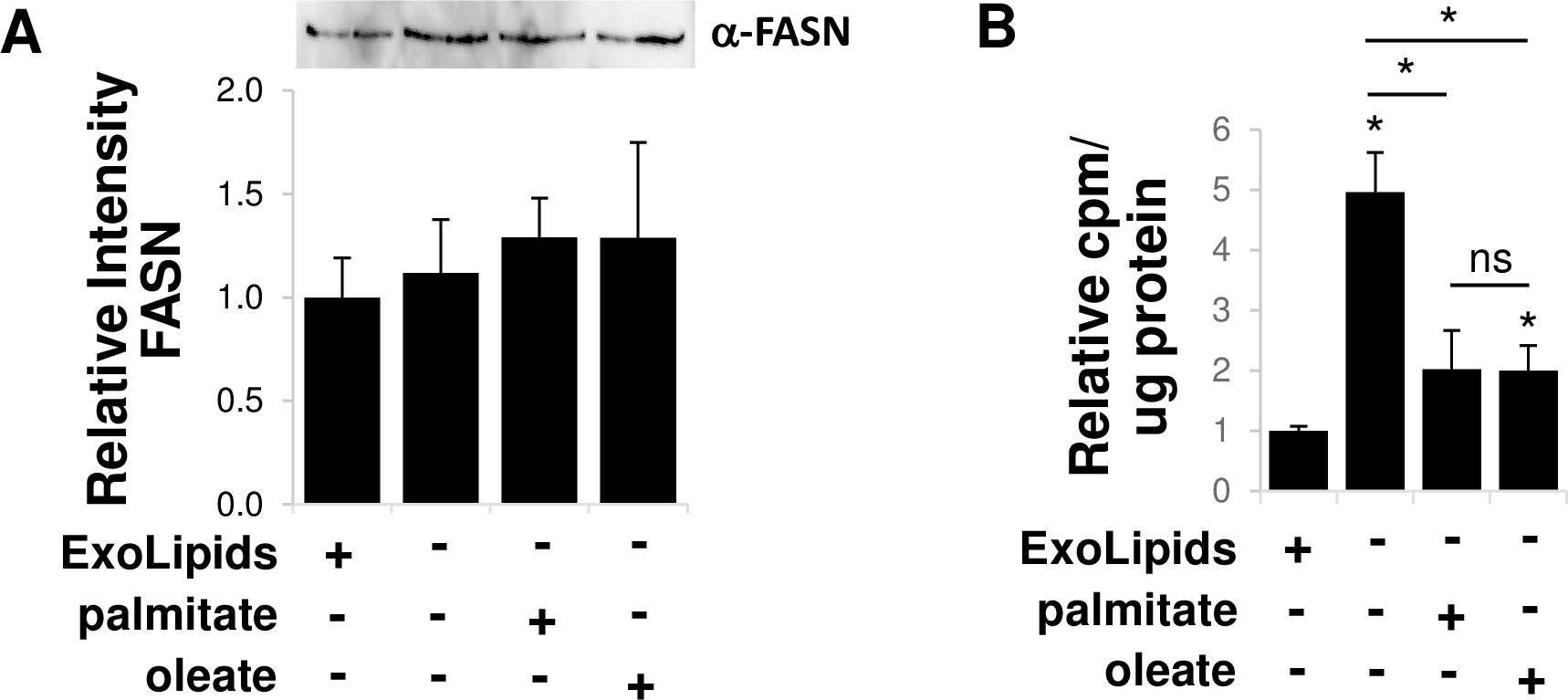

Supplement: S1 Fig — Since de novo fatty acid synthesis is regulated through SREBP in response to available lipids, one possible explanation for the differential growth of L. pneumophila in macrophages treated with oleate versus palmitate (Fig 1) was that oleate reduced fatty acid biosynthesis to a greater extent than palmitate, thereby having more pronounced effects on cellular fatty acid levels in the absence of exogenous lipid resources. To test this, FASN levels and [3H]acetate incorporation into lipids was examined under these conditions as a measure of de novo lipogenesis. A) Fatty acid treatment does not alter endogenous fatty acid synthase levels in macrophages. Primary A/J mouse macrophages were incubated in the presence (+) or absence (-) of serum (exogenous lipids, ExoLipids) or in medium lacking serum supplemented with either oleate or palmitate for 18 hrs. Cells lysates were then analyzed for FASN by Western analysis (top panel) and quantified (bottom panel), normalizing to total protein. B) Oleate and palmitate treatment similarly reduces de novo lipogenesis. Macrophage as in A) were exposed to [3H]acetate. Lipids were then extracted and examined for radiolabel incorporation, normalizing to total protein. The absence of exogenous lipids resulted in a 5-fold increase in the amount of lipid synthesis when compared to cells cultured in the presence of exogenous lipids, consistent with the response of host cells to activate lipogenesis when exogenous lipids are not available. When either palmitate or oleate was added, [3H]acetate incorporation was significantly reduced, consistent with repression of lipid biosynthetic pathways through SREBP by exogenously available fatty acids. The extent of the effect observed was similar between palmitate and oleate, indicating the differential effects of oleate and palmitate on L. pneumophila replication was not due oleate more extensively limiting de novo fatty acid synthesis in the absence of exogenous lipids. A-B) Data are the mean ± sta [file ppat.1011996.s001.tif]

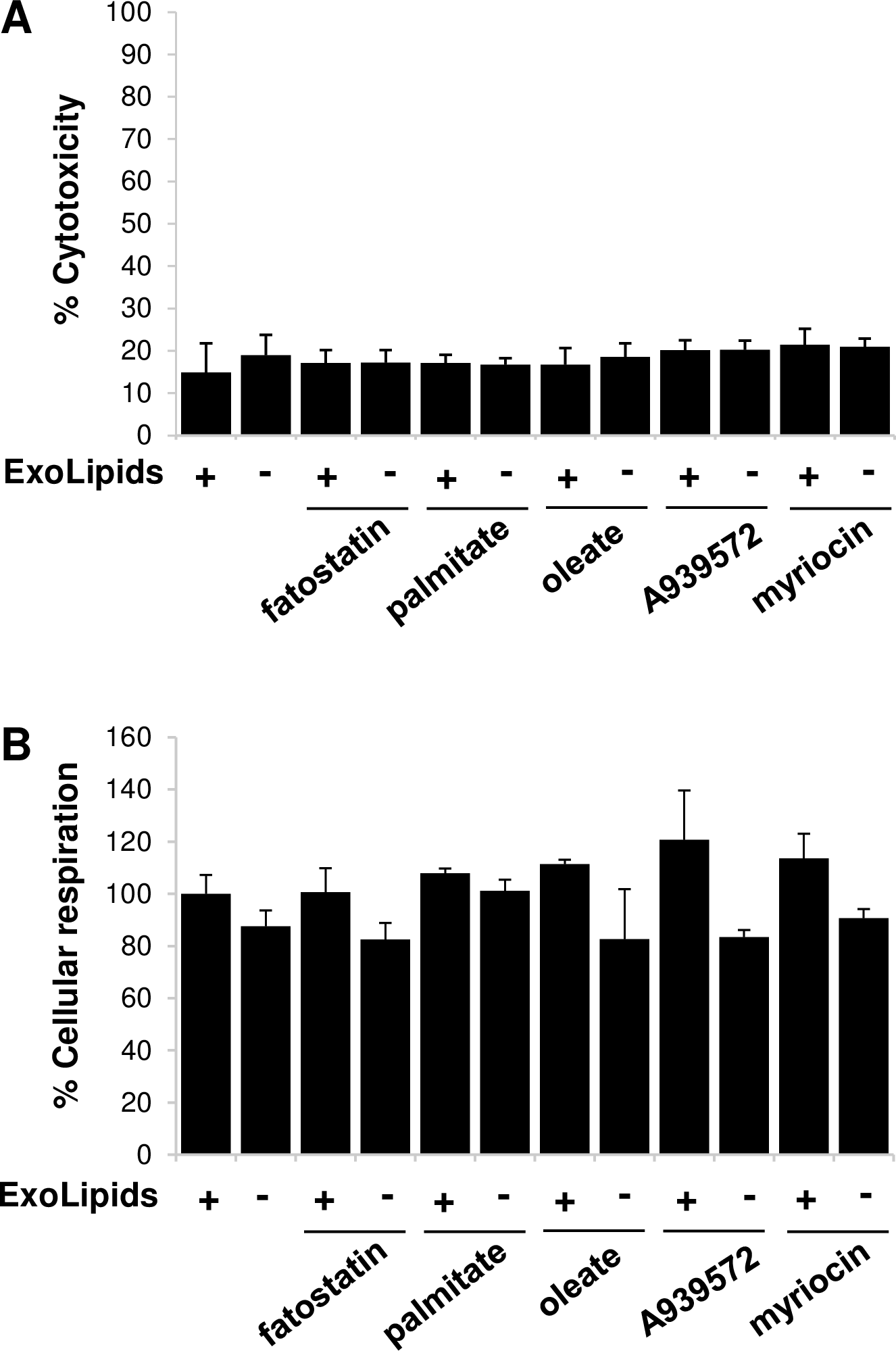

Supplement: S2 Fig — A) Primary macrophages were cultured in medium in the presence (+) or absence (-) of exogenous lipids (ExoLipids) supplemented with 4 μM fatostatin, 5 μM FAF albumin plus 20 μM palmitate or oleate, 2 μM A939572 or 100 μM myriocin for 24 hours and cell death based on lactate dehydrogenase (LDH) release was determined. B) Primary macrophages were treated as in A) and cellular respiration based on redox potential was measured. A-B) Data are the mean ± SD of 3 biological replicates, consisting of 2 technical replicates each. (TIF) [file ppat.1011996.s002.tif]

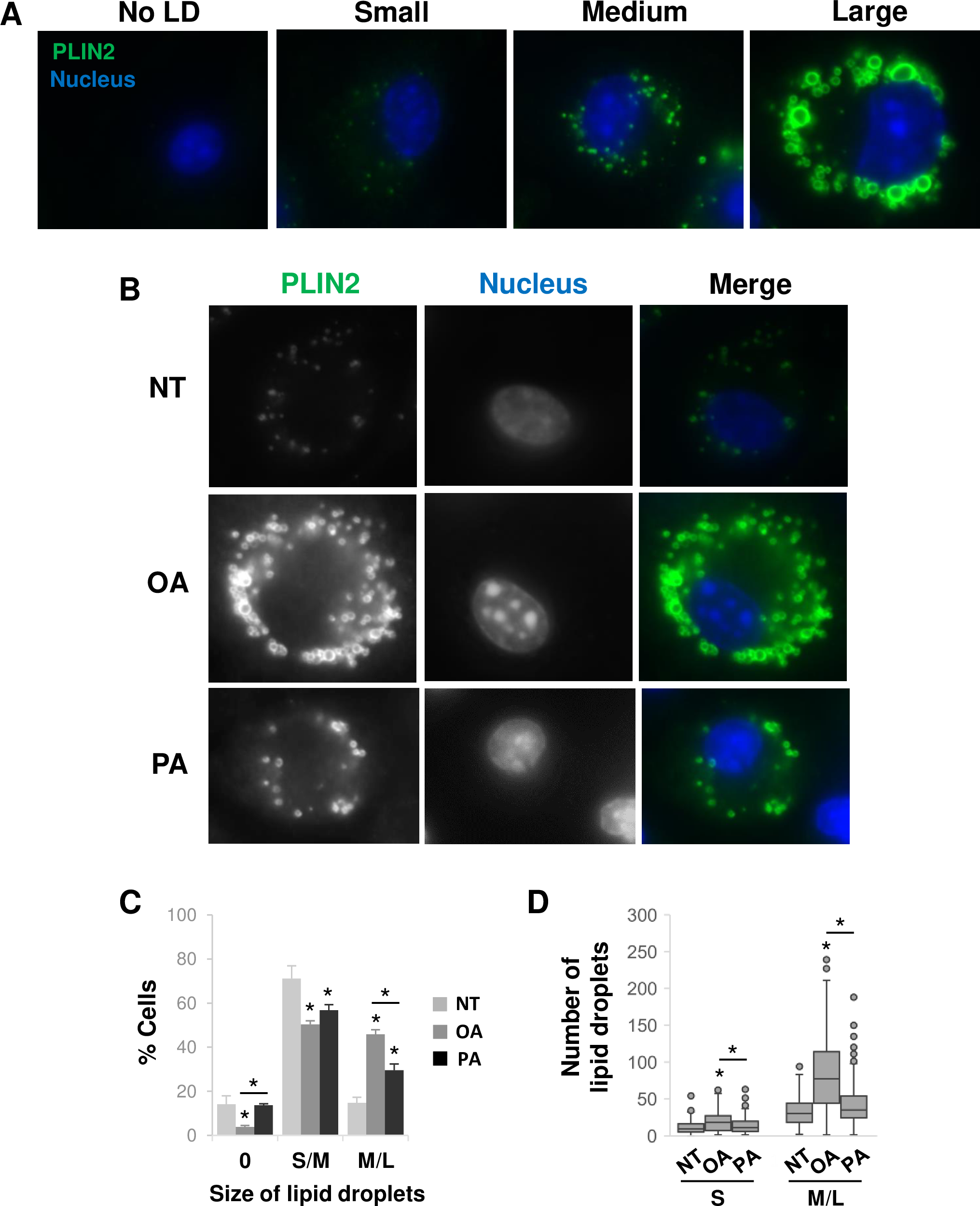

Supplement: S3 Fig — A) Differential induction of lipid droplet production in response to fatty acid treatment. Primary bone marrow-derived A/J murine macrophages were pre-treated with oleate or palmitate for 18 hrs. Cells were rinsed, fixed, stained for the lipid droplet (LD)-specific coat protein Adipose differentiation-related protein/Perilipin2 (PLIN2) [39] and visualized by fluorescence microscopy. Macrophages generally exhibited four patterns of lipid droplets: cells with no detectable lipid droplets, based on a lack of PLIN2 puncta, or cells harboring small-, medium- or large-sized lipid droplets, based on the relative diameter of the PLIN2 puncta, with some cells containing combinations of small and medium or medium and large lipid droplets. B) Pre-treating macrophages with fatty acids induced lipid droplet formation compared to no treatment (NT) control cells lacking fatty acid supplementation. C) Pre-treating cells with oleate or palmitate resulted in a higher percentage of cells harboring medium and/or large lipid droplets compared to untreated macrophages, with a more pronounced increase for oleate-treated cells relative to palmitate-treated cells. Cells in B) were scored based on the size of the PLIN2 puncta, and the percentage of cells containing no PLIN2 puncta, small or a combination of small and medium puncta (S/M), or medium and/or large puncta (M/L) was determined. Data are the mean ± standard deviation of 3 biological replicates, analyzing >100 cells per replicate. *, Student’s t-test p < 0.05 relative to NT control cells unless otherwise indicated. D) Pre-treating cells with oleate, but not palmitate, increased the number of lipid droplets, particularly in macrophages harboring medium and/or large lipid droplets. Lipid droplets based on the number of PLIN2 puncta in cells in B) were enumerated and cells containing small puncta (S) or medium and/or large puncta (M/L), were compared. Data are the mean ± standard deviation of 2 biological replicates, scoring >150 cells [file ppat.1011996.s003.tif]

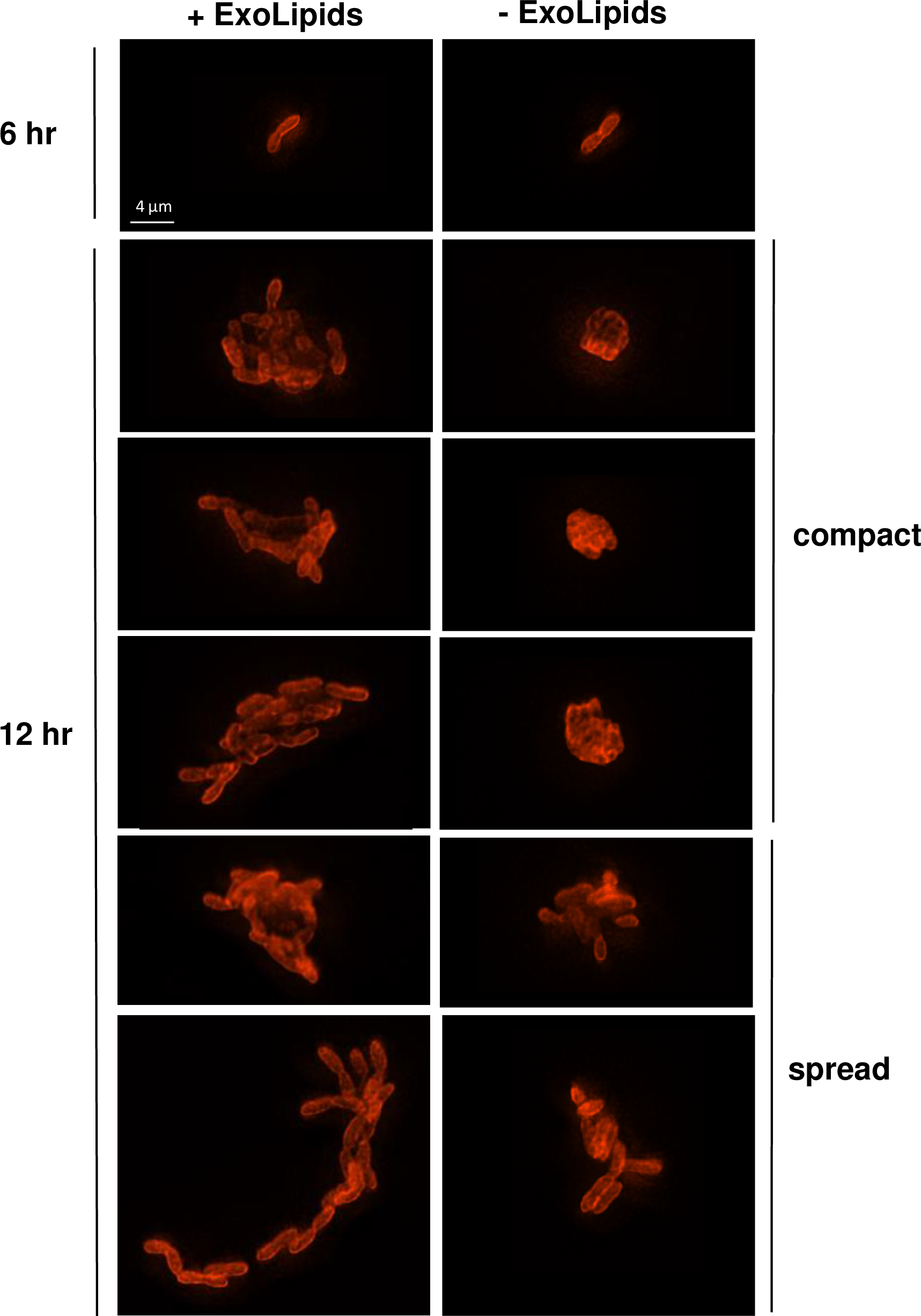

Supplement: S4 Fig — In the absence of exogenous lipids, L. pneumophila adopt a compact organization in three-dimensional space. Primary A/J mouse macrophages were infected with L. pneumophila in the presence (+) or absence (-) of serum (exogenous lipids, ExoLipids) for 6 or 12 hrs, fixed, stained and visualized by fluorescence microscopy. The organization of bacteria in the LCV in three-dimensional space was analyzed by three-dimensional deconvolution. Representative LCVs at 6 and 12 hpi from experiments in Fig 4 are shown, highlighting the typical spread organization of the LCVs when lipids are abundant (+ ExoLipids) and the combination of compact and spread LCVs observed when lipids resources are limited (- ExoLipids). (TIF) [file ppat.1011996.s004.tif]

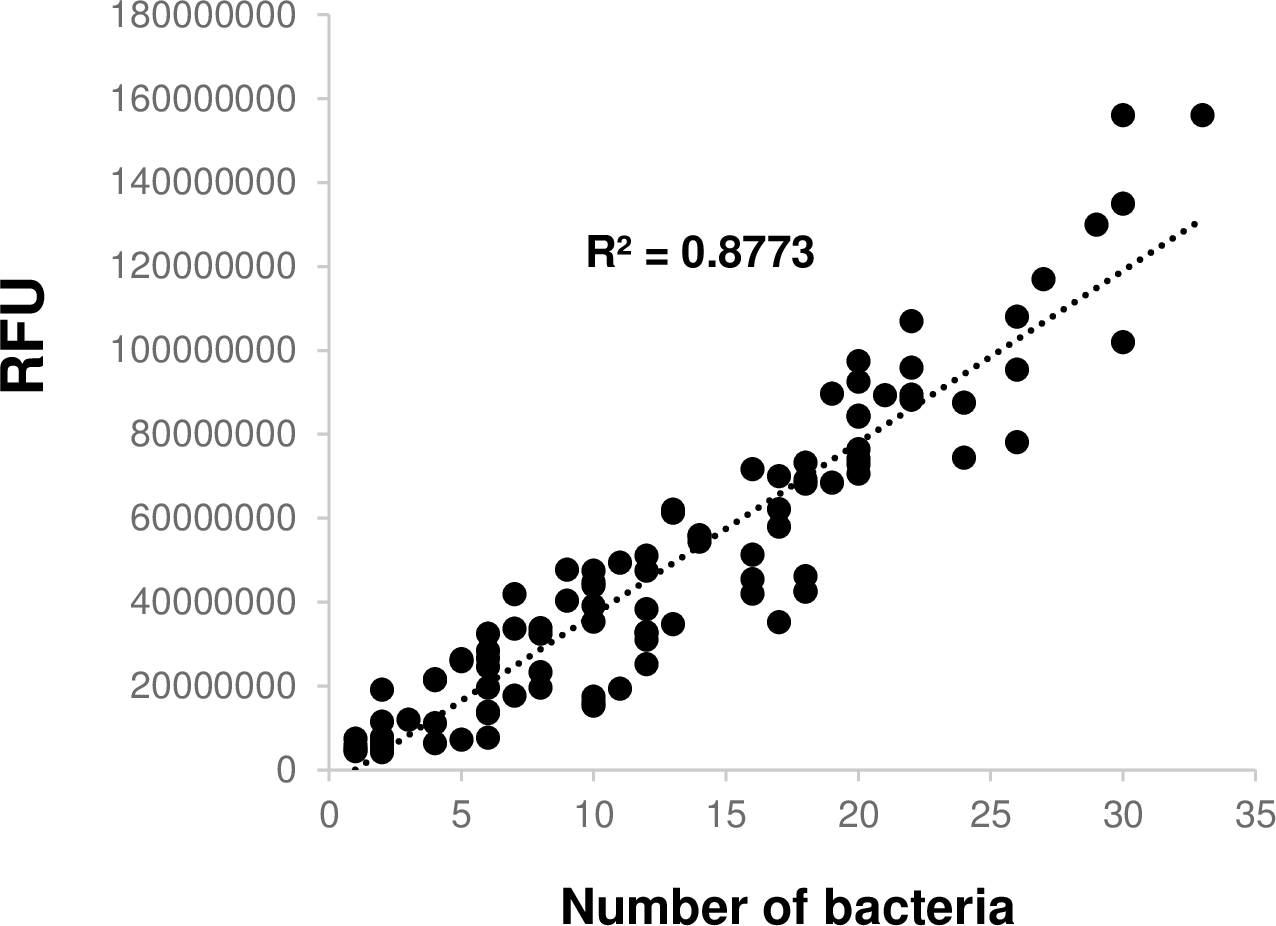

Supplement: S5 Fig — Primary A/J mouse macrophages were infected with L. pneumophila (Lp) for 12 hr. Cells were then fixed, stained and visualized by fluorescence microscopy. The fluorescence signal intensity of bacteria after immunostaining was compared to the number of bacteria counted within the LCV. RFU, relative fluorescence units. (TIF) [file ppat.1011996.s005.tif]

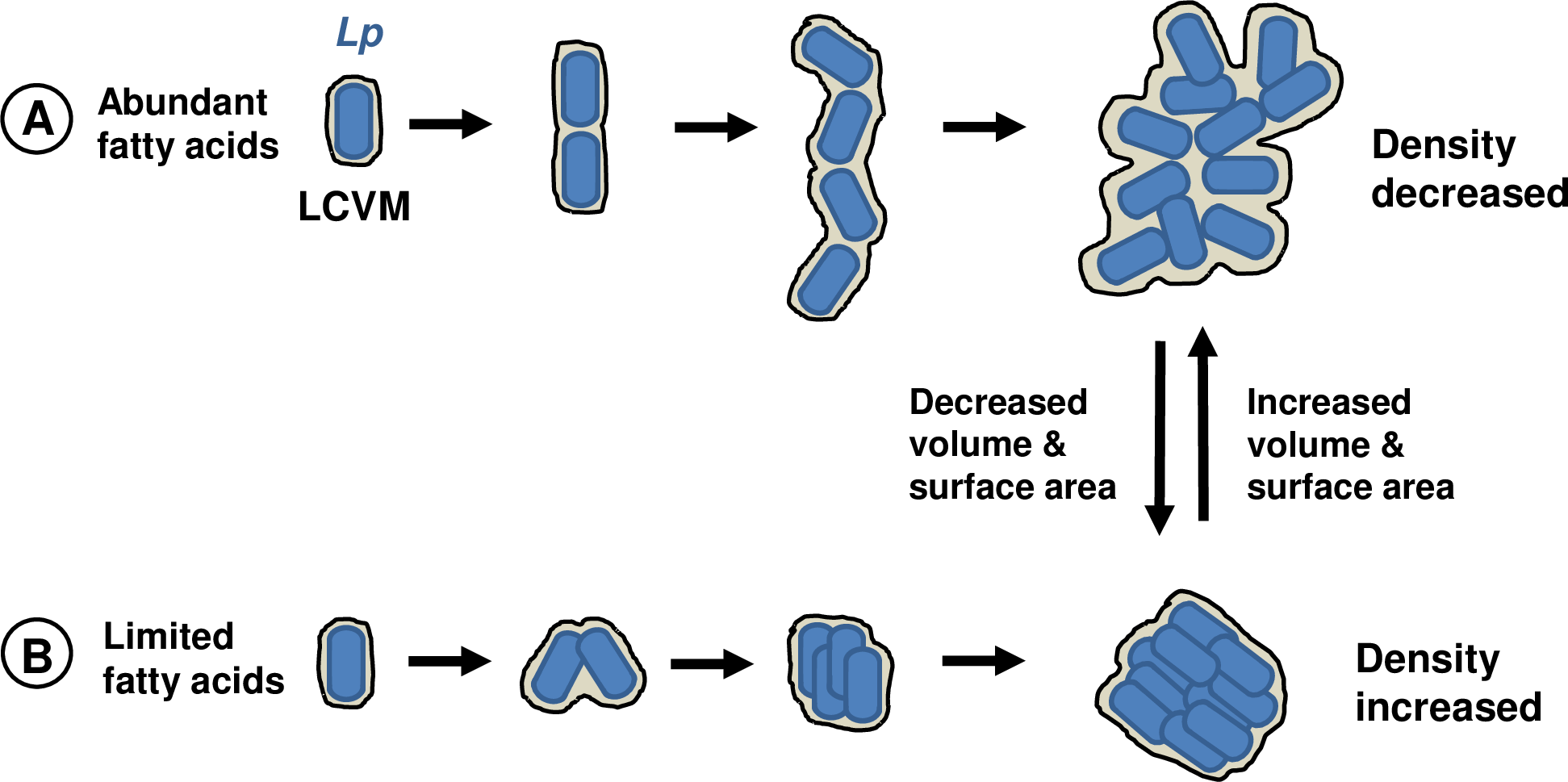

Supplement: S6 Fig — A) When host cell fatty acids resources are plentiful, the abundance of availability fatty acids to fuel LCV membrane expansion allows the bacteria spread out, adopting a spatially disordered configuration with multiple orientations in three-dimensional space. As the bacteria replicate, the volume of the LCV per bacterium increases, while the surface area required to encompass the bacteria and the space between them remains relatively constant. B) When host lipid resources become limited, LCV membrane expansion is constrained, causing bacteria to bundle together, aligning along their lengths with similar orientations in three-dimensional space. In this case, as the bacteria replicate, their close packing limits luminal space between them such that the volume of the LCV per bacteria remains constant, while adopting a more spherical shape minimizes the amount of LCV membrane required to encompass the bacteria, and thus a reduction in the surface area. The resulting compact structure maximizes volume per surface area, and thus the capacity of the LCV, allowing the maximum number of bacteria to be accommodated with the minimal amount of LCV membrane. As a consequence, compact LCVs are smaller in size but have a higher bacterial density than spread LCVs. (TIF) [file ppat.1011996.s006.tif]

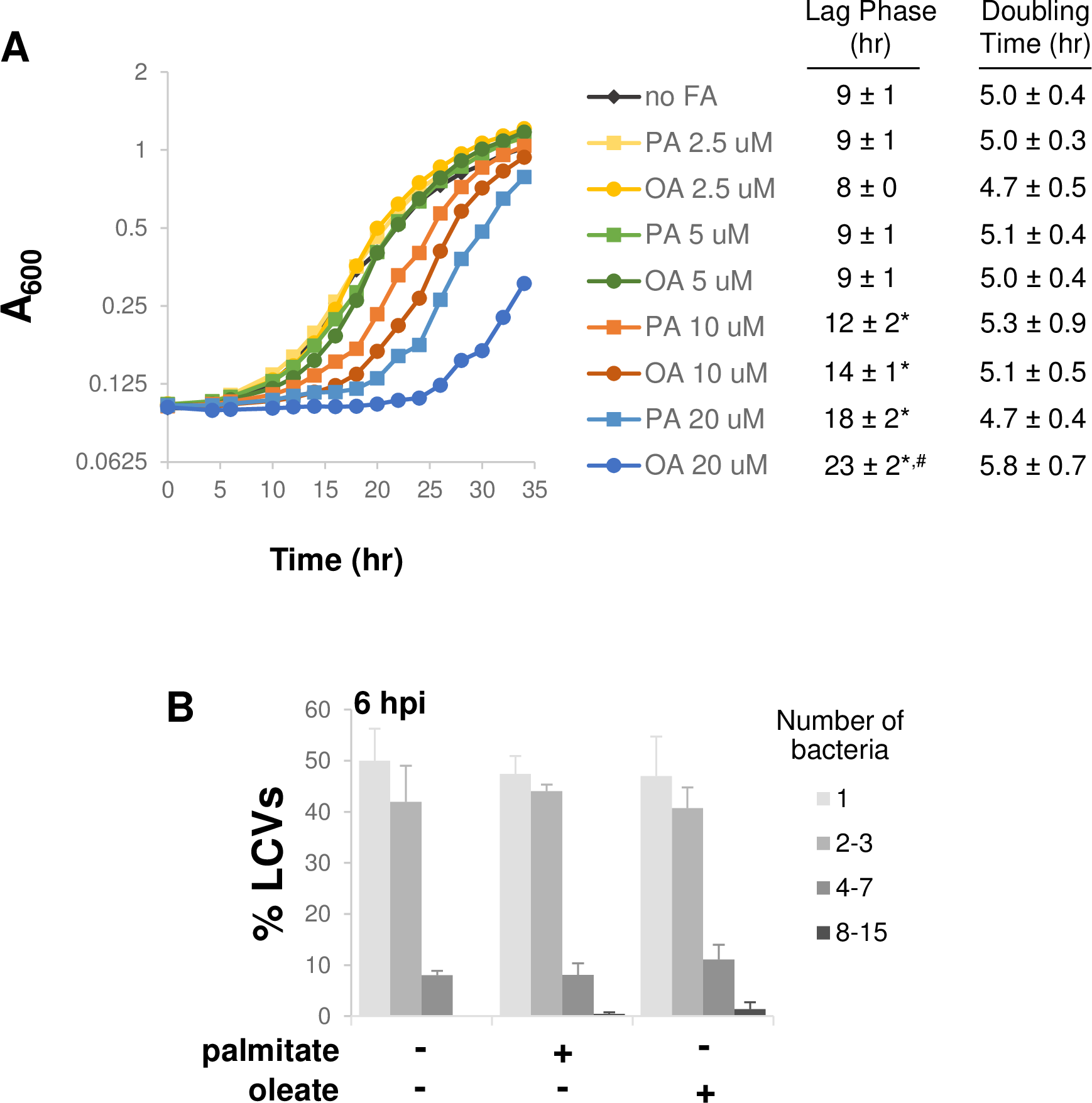

Supplement: S7 Fig — A) Exposure of L. pneumophila to fatty acids in vitro results in an extended lag phase but does not impact bacterial growth. L. pneumophila were cultured in bacteriological medium lacking fatty acids supplementation (no FA) or containing increasing amounts of either oleate or palmitate, and bacterial growth based on absorbance (A) at 600 nm (A600) was measured over time. Plotted data are representative of 3 biological replicates, which consisted of 2 technical replicates each. Reported lag phase and doubling times are the mean ± standard deviation of the 3 biological replicates represented by the data plot. *, Student’s t test p <0.05, relative to cells cultured in medium lacking fatty acid supplementation. #, Student’s t test p <0.05, relative to cells cultured in medium supplemented with the same concentration of palmitate. B) Fatty acid treatment of host cells does not impact the onset of bacterial replication during infection. Primary A/J mouse macrophages were challenged with L. pneumophila for 1 hour, rinsed and cultured in medium supplemented with palmitate or oleate and compared to cells culture in medium alone (NT, no treatment). At 6 hpi, cells were fixed, stained and visualized by fluorescence microscopy, enumerating the number of bacteria per LCV. Data are the mean ± standard deviation of 3 biological replicates, scoring >100 vacuoles per replicate. (TIF) [file ppat.1011996.s007.tif]

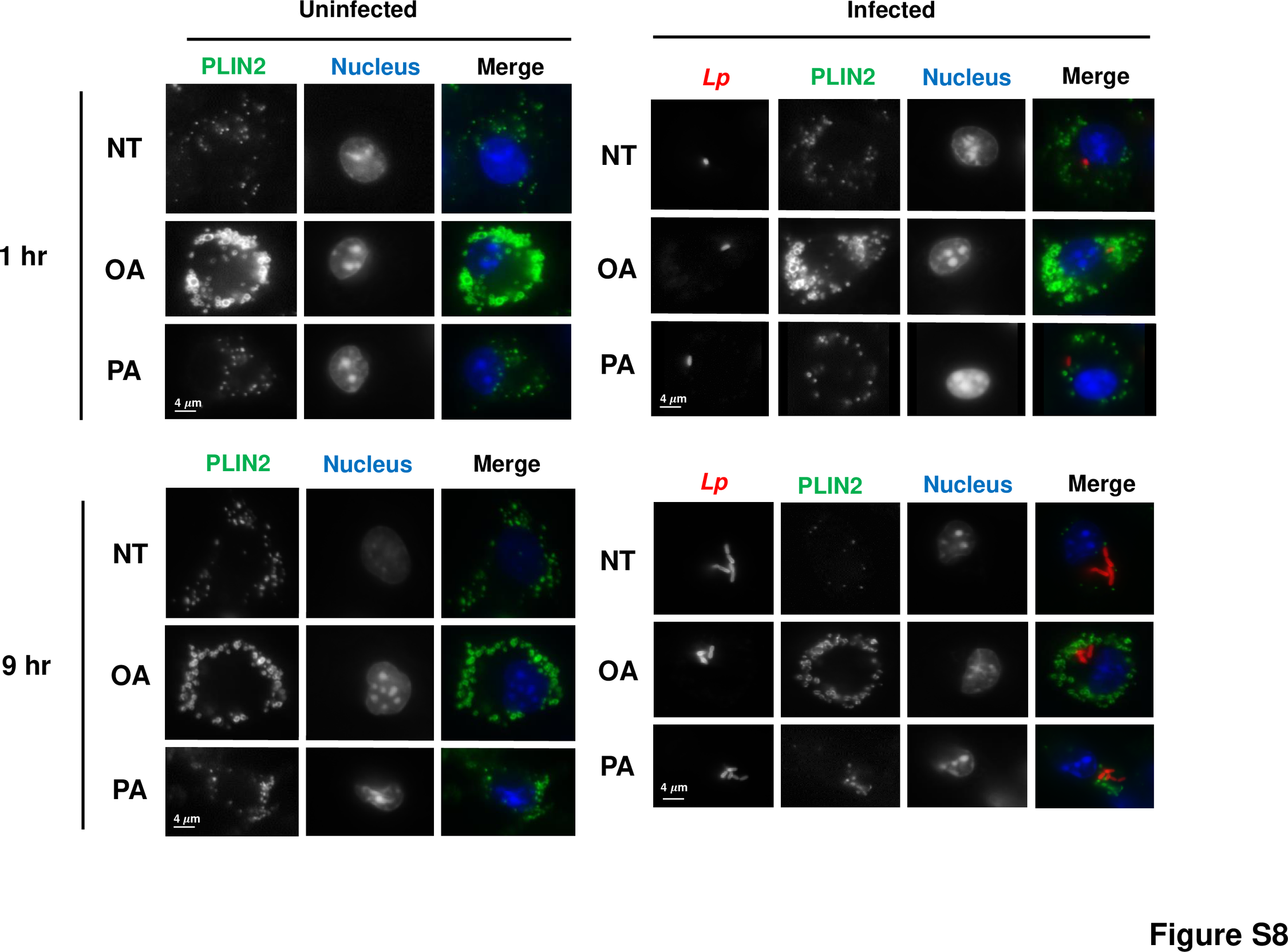

Supplement: S8 Fig — A) Primary bone marrow-derived A/J murine macrophages were pre-treated with oleate (OA) or palmitate (PA) for 18 hrs (as in S3 Fig), rinsed and then infected with L. pneumophila in the absence of exogenous lipids by culturing cells in medium lacking serum. NT, no treatment control macrophages not pre-exposed to fatty acids and cultured in the presence of serum. At 1 and 9 hpi, cells were fixed, stained for L. pneumophila (Lp), the lipid droplet coat protein Perilipin2 (PLIN2) and nuclei and visualized by fluorescence microscopy. (TIF) [file ppat.1011996.s008.tif]

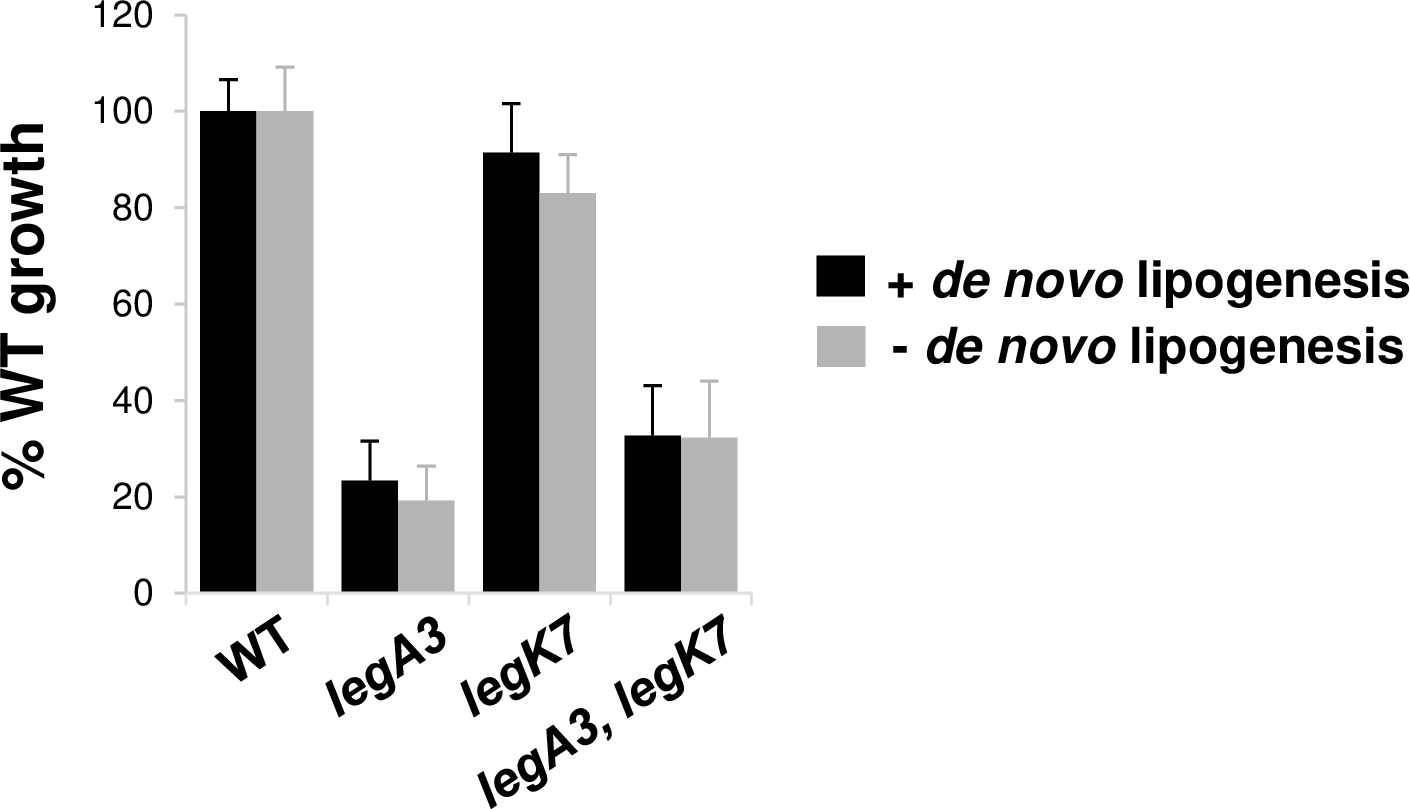

Supplement: S9 Fig — Primary bone marrow-derived A/J mouse macrophages were infected with the indicated L. pneumophila strains in the presence (+) or absence (-) of de novo lipid synthesis by treating cells with the SREBP1 inhibitor fatostatin. Bacterial growth was quantified based on recovered colony forming units (cfus) from host cell lysates at 24 hrs, and normalized to wild type (WT) bacteria in untreated host cells by the number of intracellular bacteria at 1 hpi. Data are the mean ± standard deviation of 3 biological replicates consisting of 3 technical replications each. (TIF) [file ppat.1011996.s009.tif]
